# Relief of talin autoinhibition triggers a force-independent association with vinculin

**DOI:** 10.1083/jcb.201903134

**Published:** 2019-12-05

**Authors:** Paul Atherton, Franziska Lausecker, Alexandre Carisey, Andrew Gilmore, David Critchley, Igor Barsukov, Christoph Ballestrem

**Affiliations:** 1 Wellcome Trust Centre for Cell-Matrix Research, University of Manchester, Manchester, UK; 2 Department of Biochemistry, University of Leicester, Leicester, UK; 3 Institute of Integrative Biology, University of Liverpool, Liverpool, UK

## Abstract

Talin, vinculin, and paxillin are core components of the dynamic link between integrins and actomyosin. Here, we study the mechanisms that mediate their activation and association using a mitochondrial-targeting assay, structure-based mutants, and advanced microscopy. As expected, full-length vinculin and talin are autoinhibited and do not interact with each other. However, contrary to previous models that propose a critical role for forces driving talin–vinculin association, our data show that force-independent relief of autoinhibition is sufficient to mediate their tight interaction. We also found that paxillin can bind to both talin and vinculin when either is inactive. Further experiments demonstrated that adhesions containing paxillin and vinculin can form without talin following integrin activation. However, these are largely deficient in exerting traction forces to the matrix. Our observations lead to a model whereby paxillin contributes to talin and vinculin recruitment into nascent adhesions. Activation of the talin–vinculin axis subsequently leads to the engagement with the traction force machinery and focal adhesion maturation.

## Abstract (precis)

Talin and vinculin control mechanosensing by linking adhesion receptors to the contractile actin cytoskeleton. Using a mitochondrial targeting system, Atherton et al. elucidate mechanisms regulating conformational changes required for vinculin binding to talin. Such activation mechanisms are not required for either protein to interact with the adhesion regulatory protein paxillin.

## Introduction

Focal adhesions (FAs) are sites of integrin-mediated cell adhesion to the ECM. The abundance and diversity of proteins in FAs (Horton et al., 2015) allows FAs to act as efficient signaling hubs, regulating multiple aspects of cell behavior, including migration, differentiation, and proliferation (Geiger and Yamada, 2011). Talin and vinculin are two critical regulators of the mechanical link between integrins and the actin cytoskeleton (Gauthier and Roca-Cusachs, 2018). Structurally, both talin (Goult et al., 2013a) and vinculin (Chorev et al., 2018; Cohen et al., 2005) are thought to exist in dynamic equilibrium between closed (autoinhibited) and open conformations. This has led to an attractive model in which actomyosin-mediated forces are envisaged to induce conformational changes that unmask binding sites in both proteins that support their mutual interaction and association with the contractile actomyosin machinery, plus other binding partners (Chorev et al., 2018; del Rio et al., 2009; Sun et al., 2017; Yao et al., 2014, Yao et al., 2016).

For vinculin, force is thought to overcome the strong autoinhibitory interaction (*K*_d_ ∼0.1 µM; Cohen et al., 2005) between the globular N-terminal head (domains D1–D4) and the C-terminal D5 tail domain (Vt) that masks the talin-binding site in the D1 domain (Cohen et al., 2005). Furthermore, a Förster resonance energy transfer conformation sensor has shown that vinculin is in an open conformation within FAs (Chen et al., 2005). For talin, the primary autoinhibitory interaction is between the F3 domain of the N-terminal FERM domain and R9, one of the 13 α-helical bundles (R1–R13) in the flexible C-terminal rod (Calderwood et al., 2013). Although a role for force in relieving talin autoinhibition is less clear than for vinculin, in vitro studies have clearly shown that force acting on individual talin rod domains can unmask vinculin-binding sites (VBSs) buried within their cores (del Rio et al., 2009; Yao et al., 2014), thereby facilitating vinculin binding (Carisey et al., 2013; Yao et al., 2016).

Förster resonance energy transfer–based tension sensors for both talin and vinculin show that both are under tension within FAs (Austen et al., 2015; Grashoff et al., 2010; Kumar et al., 2016; LaCroix et al., 2018), and myosin-dependent stretching of talin has been demonstrated in cells (Margadant et al., 2011). Together, these experiments suggest a model where actomyosin-mediated forces activate talin and promote vinculin binding, strengthening engagement of talin with the actomyosin machinery, which is critical for the transmission of force from the cytoskeleton to the ECM via FAs (Atherton et al., 2015, 2016; Elosegui-Artola et al., 2016; Goult et al., 2018; Sun et al., 2017). However, the idea that force induces activation of both proteins largely derives from in vitro biochemical experiments, and a clear understanding of the processes involved in talin and vinculin activation in cells requires further investigation.

The vinculin–talin axis forms a scaffold for many adhesion proteins during FA development, including the signaling protein paxillin (Carisey et al., 2013). Conversely, paxillin, which can bind to both vinculin (Deakin et al., 2012) and talin (Zacharchenko et al., 2016), is also implicated in recruiting vinculin to adhesions downstream of myosin-dependent tyrosine phosphorylation (Pasapera et al., 2010). However, to what extent paxillin binding to talin or vinculin is dependent on their activation states remains unclear.

In this study, we aimed to examine whether force is required for the initiation and stable interaction between talin and vinculin by targeting proteins to the force-free and less complex environment of the outer membrane of the mitochondria. We combined this approach with structure-based talin and vinculin point and deletion mutants to reveal the contribution of specific talin domains toward talin activation and subsequent vinculin binding and show that disrupting autoinhibition of either molecule is sufficient to induce a very stable force-independent interaction. Interestingly, the adhesion protein paxillin can be recruited to both talin and vinculin in their inactive forms independently of force, leading to a model where force-independent processes initiate an adhesion complex including integrin, talin, vinculin, and paxillin that subsequently engages the actomyosin machinery, resulting in reinforcement of this linkage.

## Results

**Figure 1.**
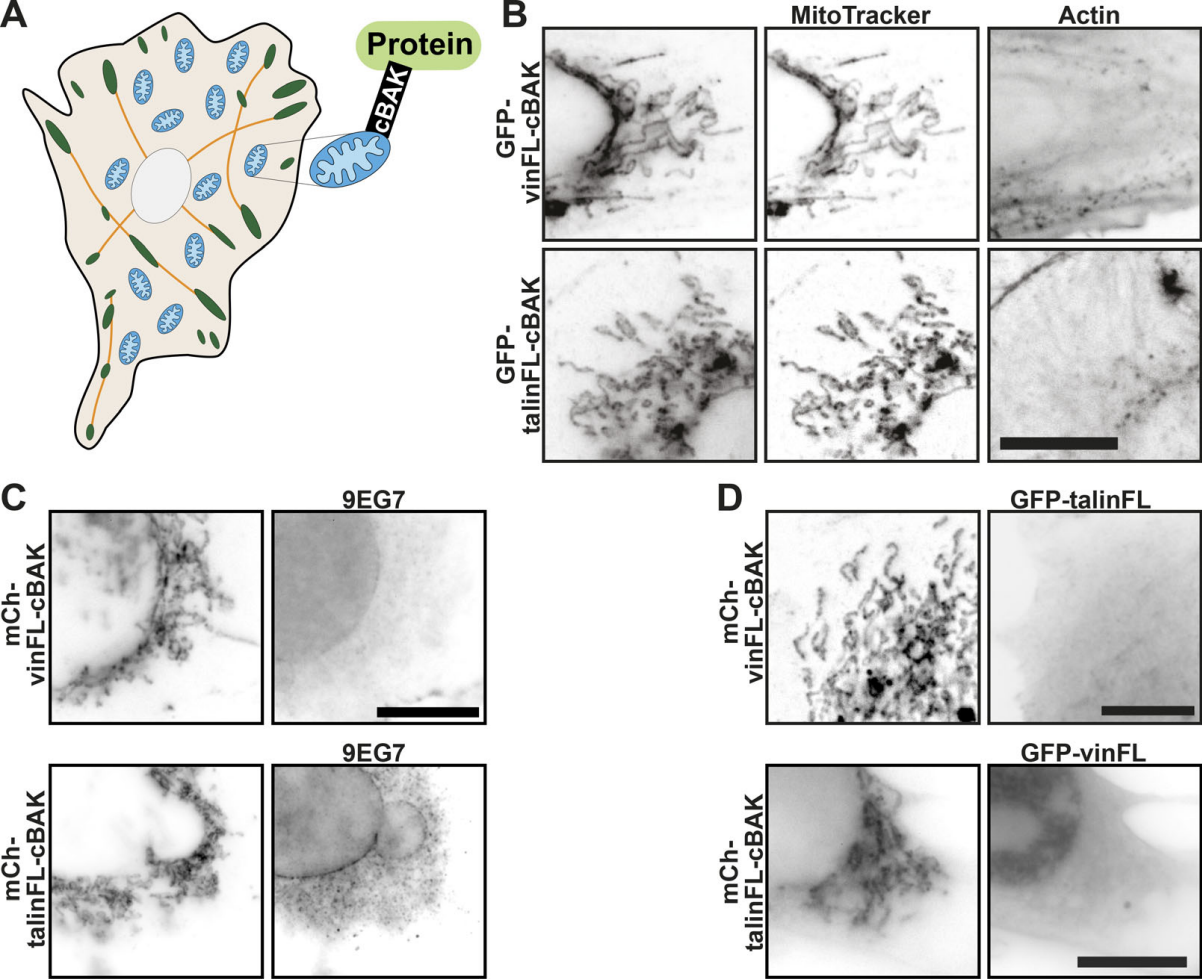
**Talin and vinculin do not interact at mitochondria. (A)** The C-terminus of talin or vinculin was fused with the short mitochondrial targeting sequence from the outer mitochondrial protein BAK (cBAK). FAs are shown in green, actin stress fibers in orange, and mitochondria in blue. **(B)** When expressed in NIH3T3 cells, vinFL-cBAK and talinFL-cBAK both colocalize with the mitochondria-specific dye MitoTracker. Phalloidin staining showed no actin at the mitochondrial surface. **(C)** Staining with 9EG7 shows that activated integrins are absent from this system. Scale bars in A–C indicate 10 µm. **(D)** Coexpression in NIH3T3 cells of GFP-talinFL and (mCherry) mCh-vinFL-cBAK, or GFP-vinFL and mCh-talinFL-cBAK, reveals that full-length vinculin and talin do not interact with each other at mitochondria.

**Figure S1.**
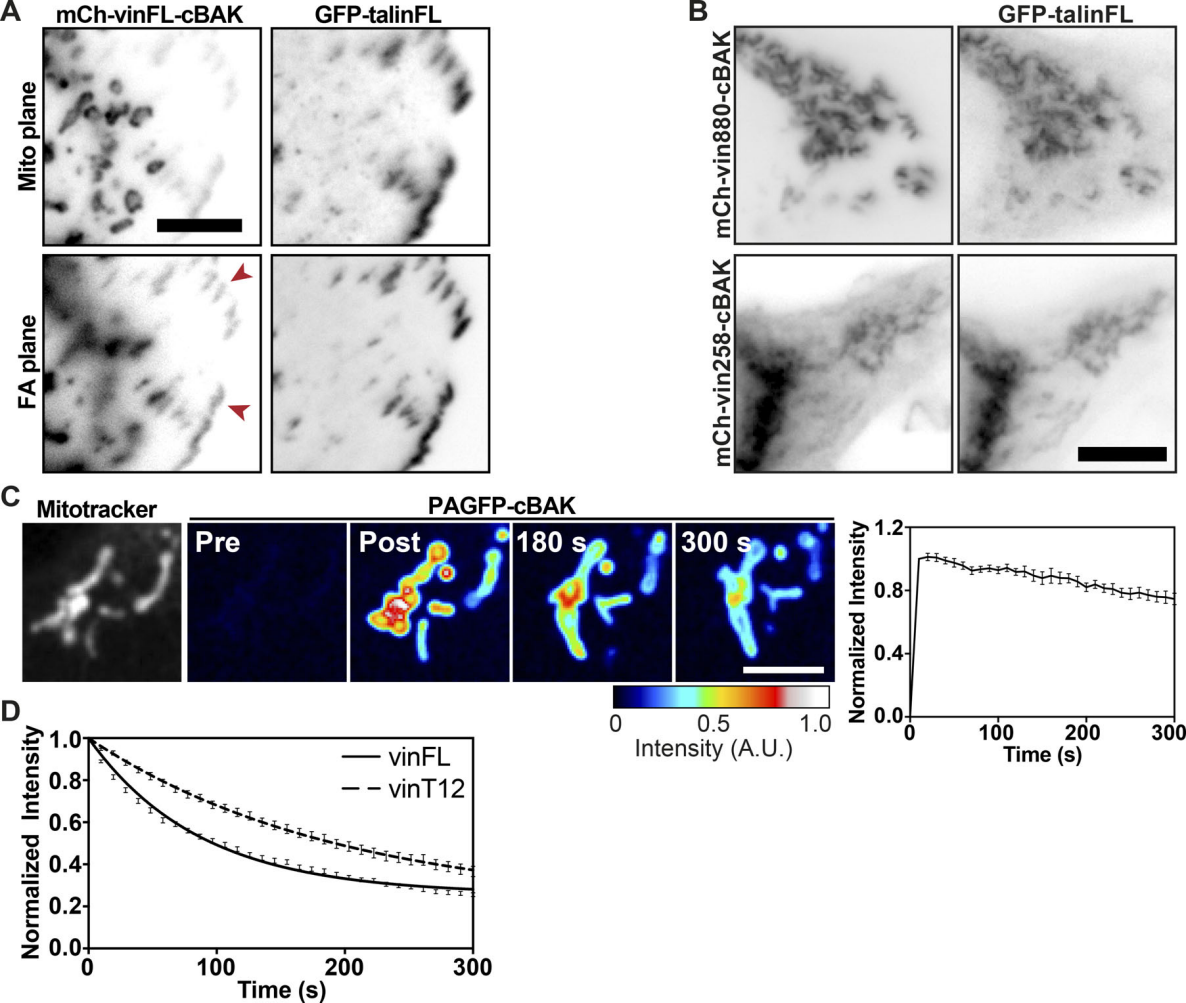
**Recruitment of talin to truncated vinculin constructs. (A) **Coexpression of GFP-talinFL and mCh-vinFL-cBAK in NIH3T3 cells. Imaging of the mitochondria plane (top panel) or the FA plane (bottom plane) shows that, while the constructs do not colocalize at mitochondria, both constructs are present at FAs (red arrows). Scale bar indicates 5 µm. **(B)** Coexpression of GFP-talinFL with either mCh-vin880-cBAK (lacking the C-terminal vinculin tail) or mCh-vin258-cBAK (the D1 domain of vinculin only) in NIH3T3 cells shows that both cBAK constructs can recruit talin. Scale bar indicates 10 µm. **(C**)**** FLAP curve and images of PAGFP-cBAK at mitochondria marked using MitoTracker Deep Red FM in NIH3T3 cells reveals that this construct stably integrates into the outer mitochondrial membrane. Scale bar indicates 5 µm; error bars represent SEM, *n* = 15 mitochondria from five cells. Results are representative of three independent repeats. **(D**)**** FLAP curves of PAGFP-talinFL at FAs coexpressed with either mCh-vinFL or mCh-vinT12. Note the reduced turnover of talin at FAs when coexpressed with vinT12. Error bars represent SEM; *n* = 92 (vinFL) or 68 (vinT12) FAs, from 10–15 cells. Data are pooled from three independent experiments.

The enormous complexity of protein–protein associations within FAs makes it virtually impossible to analyze molecular rearrangements and to separate force-dependent and force-independent processes. To overcome these limitations, and to test the role of forces in vinculin and talin activation, we fused the C-terminus of talin or vinculin with the mitochondrial targeting sequence from the C-terminus of BAK (cBAK: aa 1,072–1,162; Fig. 1 A). Colocalization with the mitochondrial-specific dye MitoTracker established successful targeting of both constructs to the mitochondria (Figs. 1 B and S1 A). Neither integrins nor F-actin was found next to mitochondria (Fig. 1, B and C), confirming a force-free environment (Fig. 1, B and C; Detmer and Chan, 2007). Crucially, neither cBAK-fused wild-type vinculin (vinFL-cBAK) nor wild-type talin (talinFL-cBAK) recruited either coexpressed or endogenous talin or vinculin, respectively (Fig. 1 D). This suggests that the C-terminal cBAK tag does not affect the structure, function or activation status of wild-type talin or vinculin and that an essential signal required for the talin-vinculin association is absent from mitochondria.

### Active vinculin binds talin without forces

**Figure 2.**
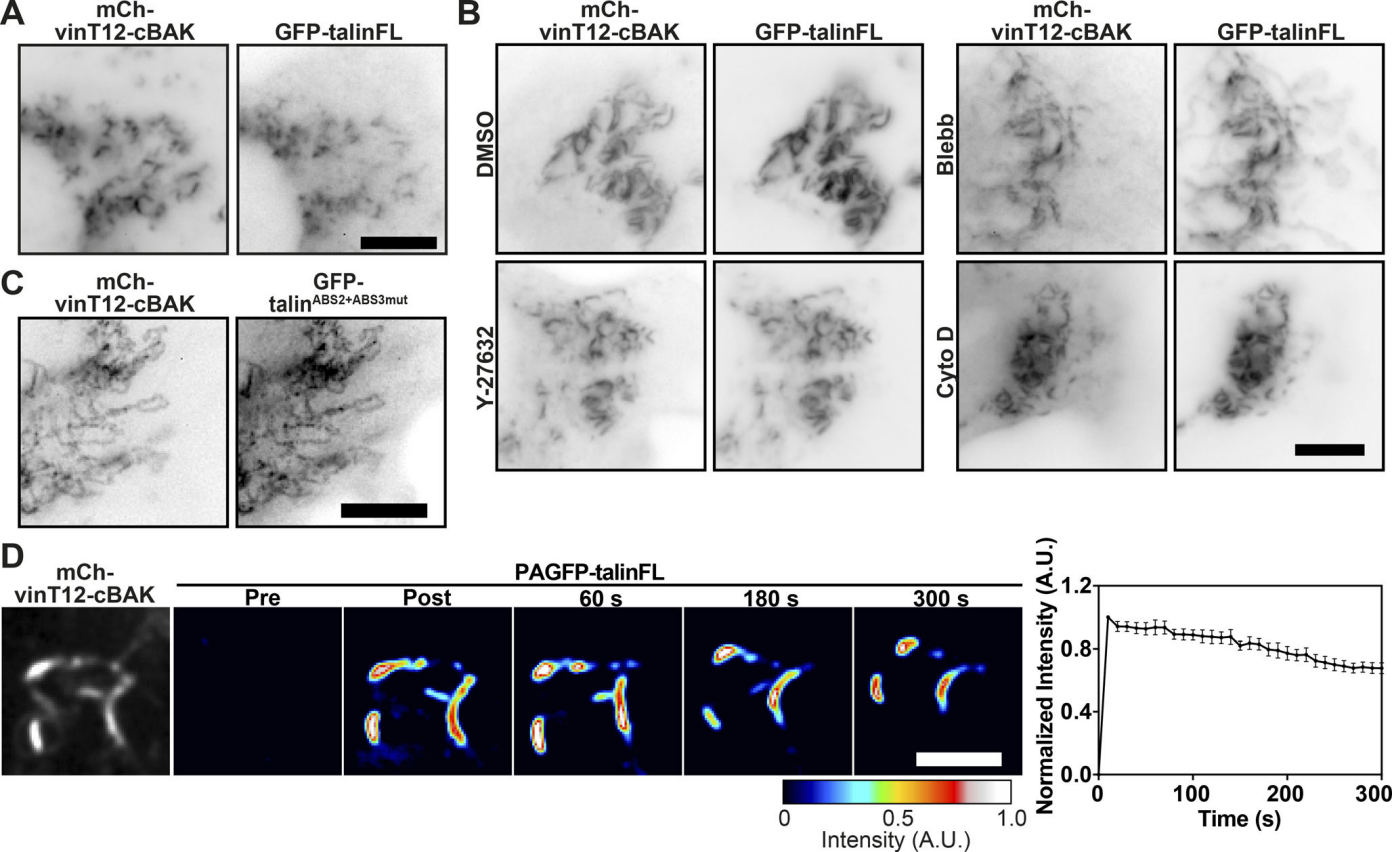
**Active vinculin can bind talin independently of force. (A)** Coexpression of active mCh-vinT12-cBAK with GFP-talinFL in NIH3T3 cells shows that the two constructs colocalize at mitochondria. **(B)** This interaction occurs in the presence of Y-27632 (50 µM), blebbistatin (50 µM), or cytochalasin D (Cyto D; 2.5 µg ml^−1^). **(C)** mCh-vinT12-cBAK also recruited a talin construct that has mutations in both actin binding sites in the talin rod (ABS2 and ABS3; GFP-talin^ABS2+ABS3mut^) in NIH3T3 cells. Scale bars in A–C indicate 10 µm. **(D)** FLAP experiments in NIH3T3 cells coexpressing mCh-vinT12-cBAK and photoactivatable (PA) GFP-talinFL show that there is minimal loss of fluorescence over time after activation, indicating a very strong interaction between the two proteins. Error bars represent SEM; *n* = 11 mitochondria from 5 cells. Results are representative of three independent experiments. Scale bar indicates 5 µm.

The lack of recruitment of vinculin to talin in the absence of force (Fig. 1 D) is in line with previously reported in vitro single-molecule stretching experiments, which concluded that the two proteins do not interact before tension being applied across talin (del Rio et al., 2009; Yao et al., 2014). Importantly, these experiments were performed using a vinculin peptide (aa 1–258) with an exposed talin-binding site, which is hidden in the full-length vinculin protein (Cohen et al., 2005). Therefore, we hypothesized that in the absence of force, talin should not interact even with a vinculin construct with an exposed talin-binding site. To test this hypothesis, we coexpressed GFP-talinFL with a constitutively active (opened) form of full-length vinculin (vinT12; Cohen et al., 2005) as well as truncated forms of vinculin (vin258 and vin880) that have exposed talin-binding sites but lack the actin-binding site located in the vinculin tail region (Carisey et al., 2013). Each vinculin construct was tagged with cBAK for mitochondrial targeting and mCherry for visualization. Surprisingly, GFP-talinFL bound to all of the vinculin constructs (Fig. 2 A and Fig. S1 B). Moreover, the interaction occurred in the presence of the actomyosin inhibitors blebbistatin or Y-27632, and also the actin polymerization inhibitor cytochalasin D (Fig. 2 B), demonstrating that actomyosin-mediated forces are not essential for talinFL to bind activated vinculin. Similarly, activated vinculin (vinT12) at mitochondria also recruited a talinFL construct bearing mutations that compromise the two actin-binding sites (ABS2 and ABS3) in the talin rod (Atherton et al., 2015; Kumar et al., 2016; Fig. 2 C).

**Figure S2.**
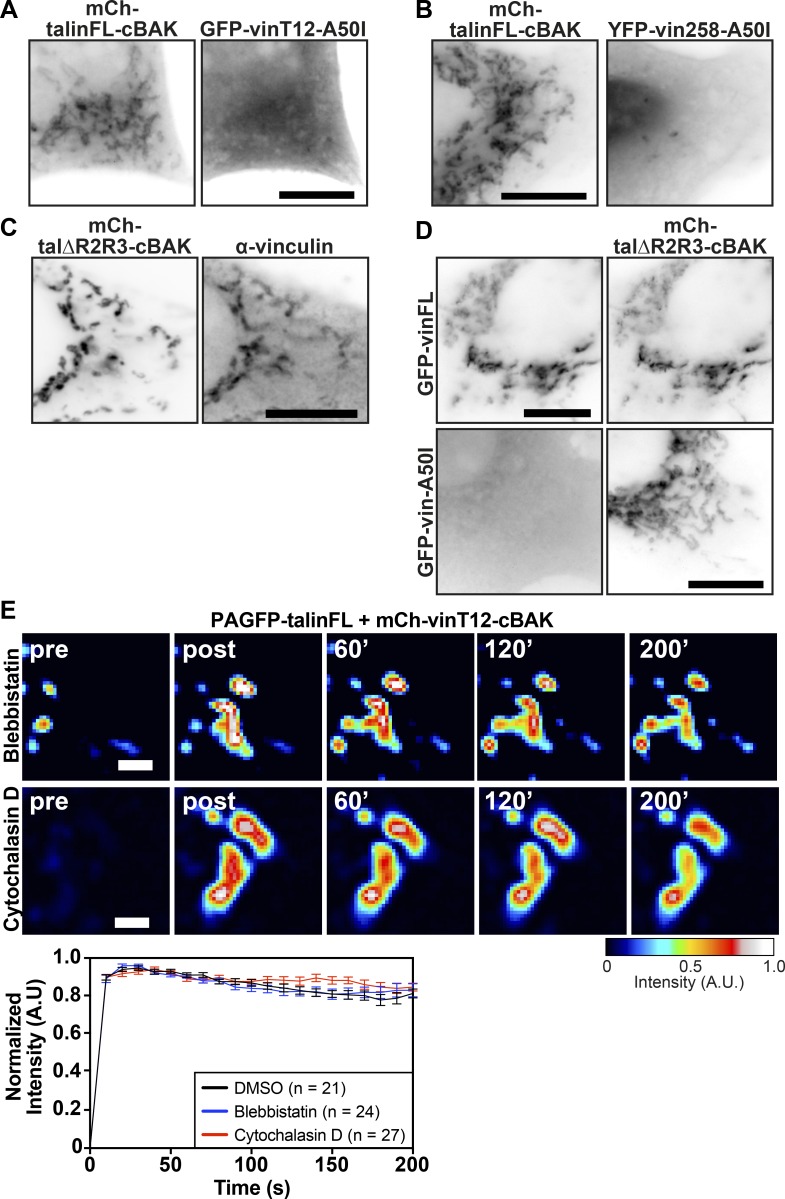
**Force-independent interactions between activated vinculin and talin, or vinculin and activated talin, are direct. (A and B**)**** Coexpression in NIH3T3 cells of mCh-talinFL-cBAK with active forms of vinculin bearing a point mutation in the canonical talin binding site in the vinculin head domain (D1) that blocks the interaction with talin: (A) GFP-vinT12-A50I or (B) YFP-vin258-A50I. **(C**)**** An activated talin deletion construct (Atherton et al., 2015) targeted to mitochondria (mCh-tal∆R2R3-cBAK) recruits endogenous vinculin (hVin1 antibody staining) in NIH3T3 cells. **(D**)**** Mutating the canonical talin binding site within the D1 domain of full-length vinculin (GFP-vin-A50I) blocks the recruitment of vinculin to active talin at mitochondria (mCh-tal∆R2R3-cBAK). Scale bars in A–D indicate 10 µm. **(E) **FLAP experiments in NIH3T3 cells coexpressing mCh-vinT12-cBAK and PAGFP-talinFL show that there is minimal loss of fluorescence over time after activation in the presence of either blebbistatin (50 µM) or cytochalasin D (2.5 µg/ml). Scale bar indicates 2 µm. Error bars represent SEM; *n* = 21 (DMSO), 24 (Blebbistatin), and 27 (Cytochalasin D) mitochondria per cell; N = 5 (DMSO), 7 (Blebbistatin), and 5 (Cytochalasin D) cells; results are representative of three independent repeats.

In FAs, increased engagement of talin and vinculin with the actomyosin machinery has been proposed to induce conformations that lead to their activation and thus reduce their mobility (Elosegui-Artola et al., 2016; Humphries et al., 2007). Hence, we speculated that the binding of activated/truncated forms of vinculin to talinFL at mitochondria (a site lacking the forces proposed to unmask binding sites in talin and vinculin) might be of low affinity, resulting in high turnover rates. However, fluorescence loss after photoactivation (FLAP) experiments revealed that, similar to the turnover of PAGFP-cBAK (Fig. S1 C; Schellenberg et al., 2013), the interaction between talinFL and active vinculin at mitochondria is extraordinarily stable (Fig. 2 D), with mobile fractions (Mfs) <20%, and remained similar in presence of blebbistatin or cytochalasin D (Fig. S2 E). Interestingly, the turnover of talinFL bound to vinT12-cBAK at mitochondria was slower than talinFL bound to vinT12 at FAs (Fig. S1 D). We conclude that vinculin constructs that already have an exposed talin-binding site can activate talinFL and bind to it with high affinity without the involvement of force. This explains the presence of stable force-independent adhesions in the presence of activated vinculin (Atherton et al., 2015; Carisey et al., 2013).

### Active talin disrupts vinculin head–tail autoinhibition

**Figure 3.**
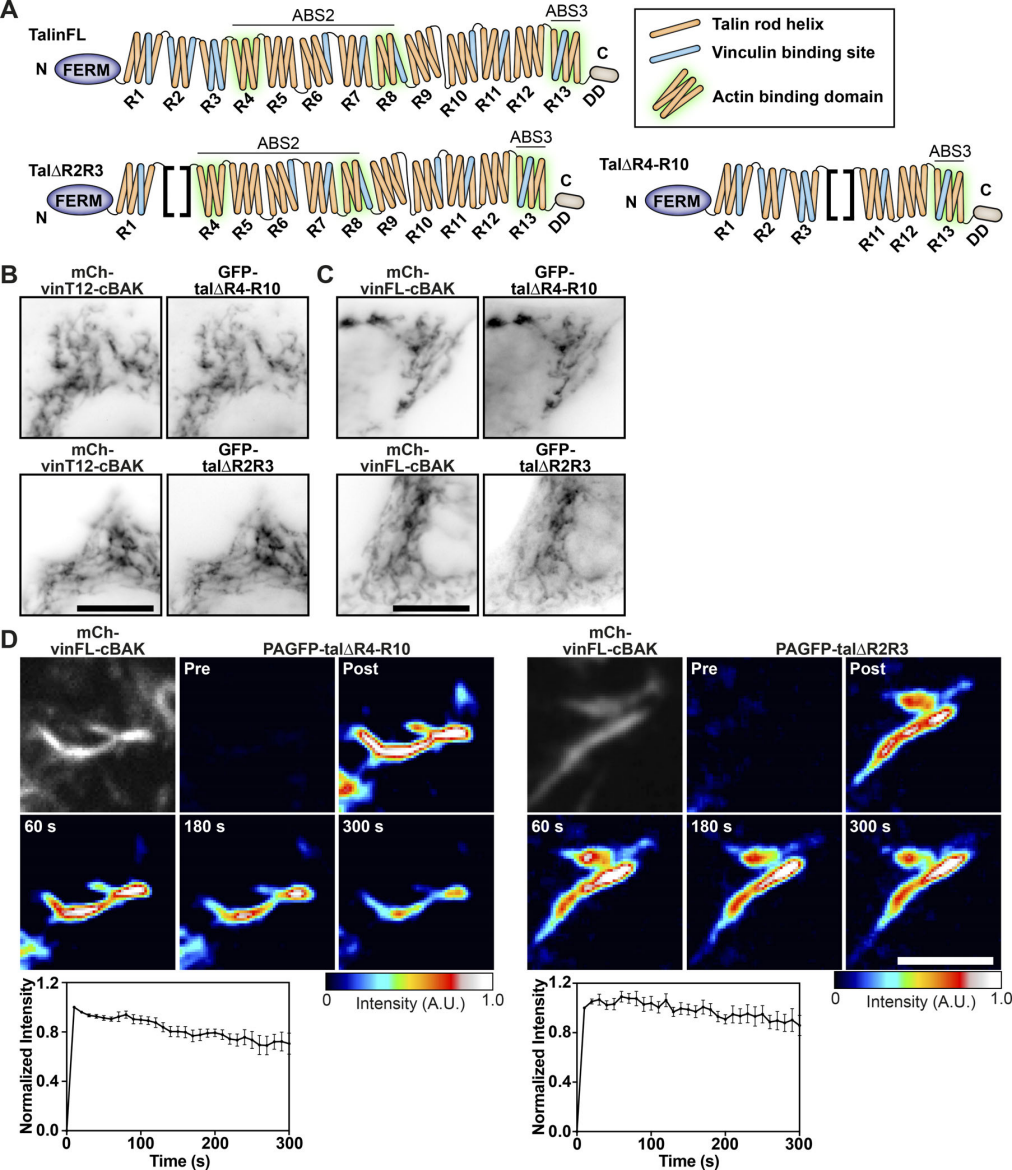
**Active talin can bind to vinculin independently of force. (A)** Schematics of the talin constructs used. Blue indicates VBSs within the four-and five-helix bundles that make up the talin rod (R1–R13), and the green halo indicates the rod domains involved in binding F-actin. The position of the talin rod domains deleted in talΔR2R3 and talΔR4-R10 are indicated by brackets. **(B and C)** GFP-talΔR4-R10 and GFP-talΔR2R3 are both recruited to either constitutively active mCh-vinT12-cBAK (B) or wild-type mCh-vinFL-cBAK (C) when coexpressed in NIH3T3 cells. Scale bars indicate 10 µm. **(D)** FLAP experiments in NIH3T3 cells show that there is minimal loss of fluorescence over time, indicating that the interaction between vinFL-cBAK and talΔR4-R10 (upper panel) and talΔR2R3 (lower panel) is very stable. Error bars represent SEM; *n* = 8 (PAGFP-talΔR2R3) or 6 (PAGFP-talΔR4-R10) mitochondria from five cells. Results are representative of three independent experiments. Scale bar indicates 5 µm.

There are numerous potential VBSs throughout the rod (Gingras et al., 2005; Fig. 3 A), and we next aimed to determine which regions of the talin rod can interact with vinculin in the absence of force. To this end, we coexpressed talin constructs lacking domains R4–R10 (GFP-talΔR4-R10) or R2–R3 (GFP-talΔR2R3; Fig. 3 A) together with constitutively active vinT12-cBAK. Both talin constructs colocalized with vinT12-cBAK at mitochondria (Fig. 3 B), demonstrating that VBSs within the N-terminal (R1–R3) and more C-terminal (R4–R13) regions of the talin rod are able to bind activated vinculin independent of force. Surprisingly, these talin deletion constructs also bound to coexpressed wild-type vinFL-BAK (Fig. 3 C). A mutation in the talin-binding site of vinculin (A50I; Bakolitsa et al., 2004) blocked the recruitment of vinculin to talin at mitochondria (Fig. S2), demonstrating that these talin–vinculin interactions at mitochondria are mediated by the canonical pathway (Bakolitsa et al., 2004). Moreover, FLAP experiments showed that these interactions between vinFL-cBAK and the talin truncation mutants had a similar stability to the interaction between talinFL and vinT12-cBAK (Fig. 3 D).

From these experiments, we conclude that the talin rod contains at least two domains, one in the R2R3 and one in the R4–R10 region, that can disrupt the vinculin head–tail interaction, leading to vinculin activation in a force independent manner. The lack of interaction between vinFL and talinFL clearly establishes that the vinculin-activating domains in talin are not accessible in the autoinhibited talin structure.

### Relief of talin autoinhibition is sufficient to induce vinculin binding

**Figure 4.**
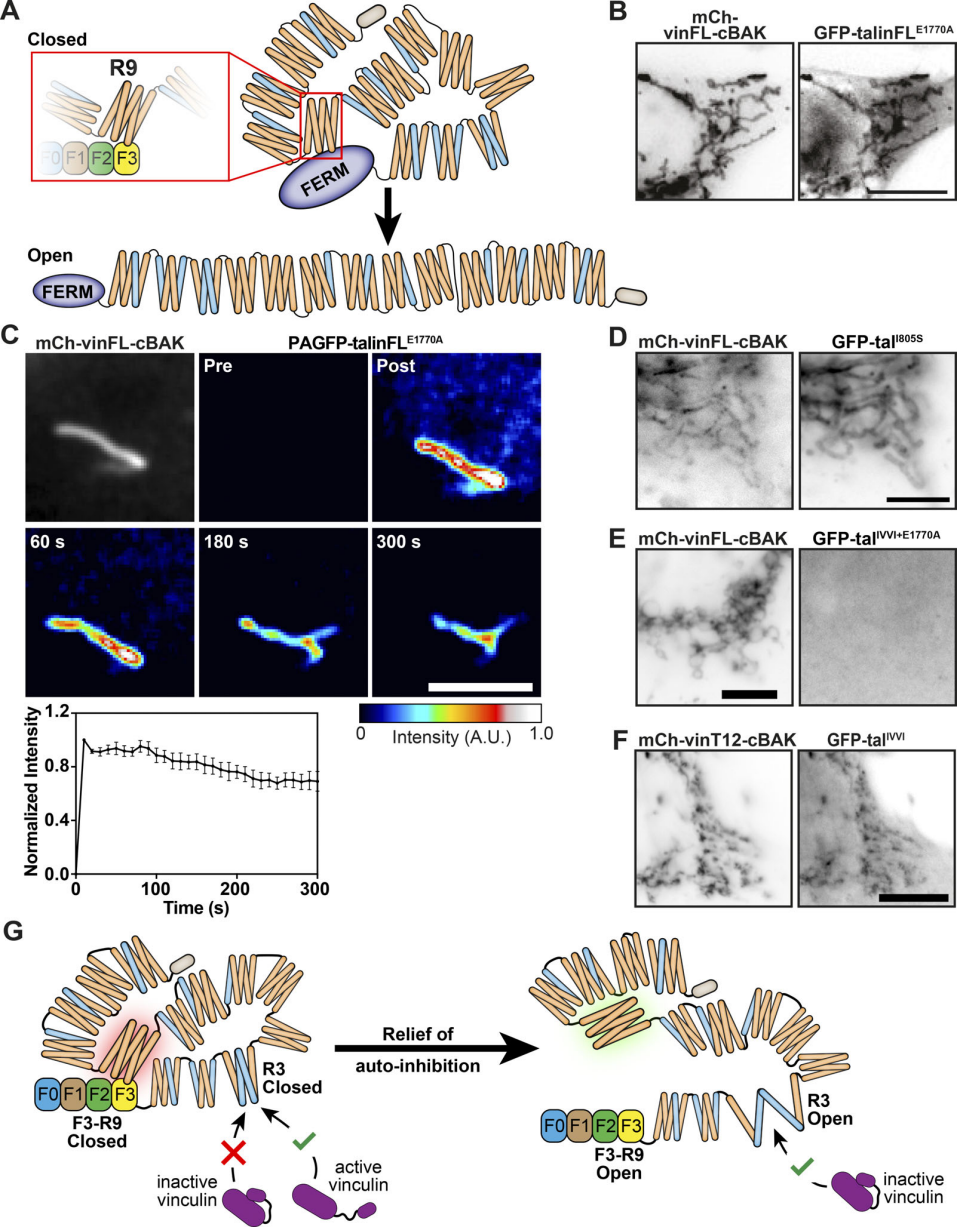
**The talin R3 domain is a critical regulator of vinculin binding and talin activation in response to disruption of talin autoinhibition. (A)** Schematic showing talin in a closed compact conformation where the R9 rod domain interacts with F3 of the FERM domain and an open talin conformation. Blue helices indicate VBSs. **(B)** GFP-talinFL^E1770A^ (autoinhibition relieved) is recruited to mCh-vinFL-cBAK when coexpressed in NIH3T3 cells. Scale bar indicates 10 µm. **(C)** FLAP experiments in NIH3T3 cells shows there is minimal loss of fluorescence over time of photoactivated PAGFP-talinFL^E1770A^ when bound to mCh-vinFL-cBAK at mitochondria. Scale bar indicates 5 µm. Error bars show the SEM; *n* = 7 mitochondria from five cells. Results are representative of three independent experiments. **(D)** GFP-talFL^I805S^ (an R3-destabilizing mutant) is even recruited to wild-type mCh-vinFL-cBAK at mitochondria. **(E)** A GFP-talin construct in which F3-R9 autoinhibition is relieved but R3 is stabilized (GFP-tal^IVVI+E1770A^) is not recruited to wild-type mCh-vinFL-cBAK at mitochondria. **(F)** GFP-tal^IVVI^ (R3 rod domain–stabilizing mutant) is capable of binding to constitutively active mCh-vinT12-cBAK. Experiments in D–F were all performed in NIH3T3 cells, and scale bars indicate 10 µm. **(G)** Model explaining how relief of talin autoinhibition regulates the potential for vinculin to bind to the R3 rod domain of talin. In the closed, autoinhibited talin conformation, only active vinculin is capable of binding to talin R3. When the F3-R9 autoinhibition is relieved, the R3 domain undergoes a conformational change, allowing wild-type vinculin to bind.

**Figure S3.**
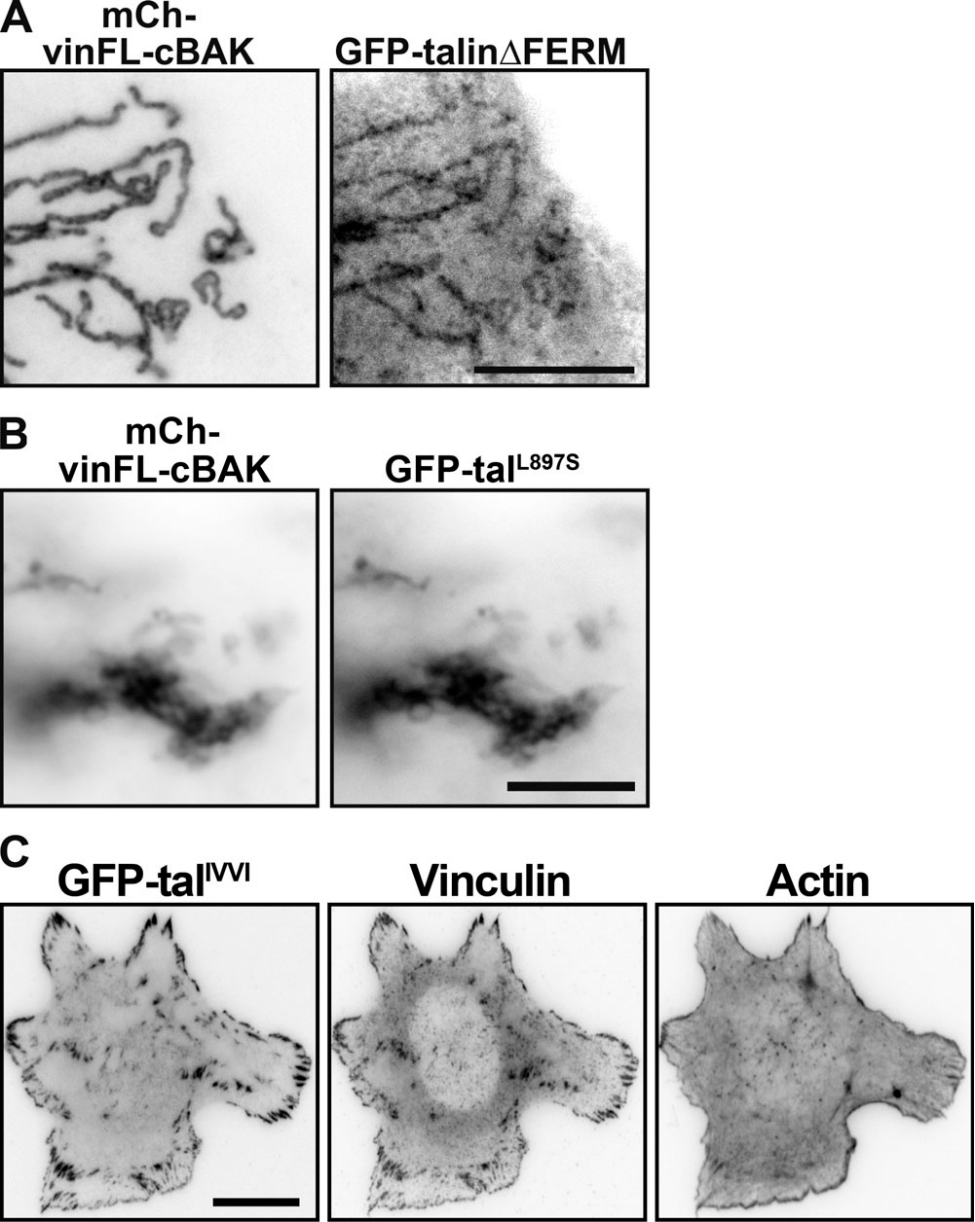
**Localization of talin^IVVI^ to FAs in **t**alinKO cells. (A**)**** Coexpression of a GFP-talin∆FERM construct with mCh-vinFL-cBAK in NIH3T3 cells reveals this construct can bind to inactive vinculin. Scale bar indicates 10 µm. **(B**)**** Coexpression of GFP-tal^L897S^ with mCh-vinFL-cBAK in NIH3T3 cells reveals that destabilizing the R3 helical bundle through this point mutation (Rahikainen et al., 2017) permits talin–vinculin binding. Scale bar indicates 10 µm. **(C)** Expression of GFP-tal^IVVI^ (containing R3 stabilizing mutations [Goult et al., 2013b]) in talinKO cells shows this construct localizes to FAs.

The current model of talin (Fig. 4 A) suggests the actin and VBSs are unavailable in cytoplasmic form of the molecule (Goult et al., 2013a). Previous studies suggested disruption of F3–R9 autoinhibition as an early step in talin activation (Goksoy et al., 2008) that is required for integrin binding and activation (Goksoy et al., 2008; Goult et al., 2009). We questioned whether disrupting this F3–R9 interaction would promote the conformational changes required to permit force-independent vinculin binding. To test this hypothesis, we introduced an E1770A mutation in the talin R9 domain (talin^E1770A^) that disrupts the talin F3–R9 interaction, thus relieving talin autoinhibition (Fig. 4 A; Goult et al., 2009). In contrast to wild-type talin, the talin^E1770A^ mutant readily bound to vinFL-cBAK (Fig. 4 B), and FLAP experiments demonstrated that this interaction was very stable (Mf < 20%; Fig. 4 C). Similarly, a talin ΔFERM rod only construct (Wang et al., 2011) also bound vinFL-cBAK (Fig. S3 A). These findings demonstrate that disrupting the F3–R9 interaction is sufficient to expose vinculin-activating domains in the talin rod.

### The R3 domain of talin is a key determinant of vinculin binding

Vinculin binding to the talin R2/R3 rod domains triggers a conformational change that regulates actin binding to the adjacent R4–R8 domains (ABS2; Atherton et al., 2015). The experiments above suggest that relief of talin autoinhibition could induce similar conformational changes in talin permitting vinculin binding. In vitro experiments indicate that the talin R3 four-helix bundle, which contains two VBSs, is the first helical bundle to unfold in response to force (Yao et al., 2014), and high-pressure nuclear magnetic resonance experiments show that this domain is inherently unstable, existing in equilibrium between closed and partly open states (Baxter et al., 2017). This is largely due to a cluster of four threonine residues buried within the hydrophobic core of R3. Thus, we hypothesized that introducing additional hydrophilic residues into the R3 core (tal^I805S^ or tal^L897S^) that shift the equilibrium toward the open state (Rahikainen et al., 2017) may on their own be sufficient to expose VBS in talin and thereby activate vinculin. Indeed, we found that such mutations triggered binding of mutant talinFL to autoinhibited vinFL-cBAK (Figs. 4 D and S3 B).

Conversely, we hypothesized that increasing the stability of R3 would have the opposite effect. Mutating the four threonine residues buried within the R3 core to hydrophobic residues (T809I, T833V, T867V, and T901I; GFP-tal^IVVI^) has previously been shown to stabilize the R3 helical bundle (Yao et al., 2014), requiring more force to stretch this domain (Goult et al., 2013a; Yao et al., 2014). Since talinFL and vinFL do not interact at mitochondria unless one of the two is active, we introduced the IVVI stabilizing mutations into the talin^E1770A^ mutant in which autoinhibition is relieved. Remarkably, we detected no interaction between the GFP-talFL^IVVI-E1770A^ double mutant and vinFL-cBAK (Fig. 4 E) in marked contrast to the strong and stable association of the GFP-talin^E1770A^ mutant with vinFL-cBAK at mitochondria. This result is important, since it suggests that after relief of talin autoinhibition, rearrangements in the R3 domain are absolutely critical for binding to vinFL and the exposure of the talin-binding site in the vinculin head. However, to our surprise, a GFP-tal^IVVI^ construct was recruited to activated vinT12-cBAK (Fig. 4 F) at mitochondria, as well as to the vin258-cBAK construct (vinD1 domain only; not shown). These results show that active vinculin can even bind to and activate autoinhibited talin containing a stabilized R3 domain.

In summary, these results lead to the following model (Fig. 4 G). Initially, activated talin (after relief of autoinhibition) binds to vinFL via the two VBSs within the talin R3 helical bundle. Binding to these VBSs is sufficient to activate vinculin by disrupting the vinculin head–tail interaction, thus facilitating further vinculin binding to talin. Once activated, vinculin (with an exposed talin-binding site) can then bind to talinFL at multiple VBSs within the talin rod independent of force.

### Talin, vinculin, and force-independent FA assembly

**Figure 5.**
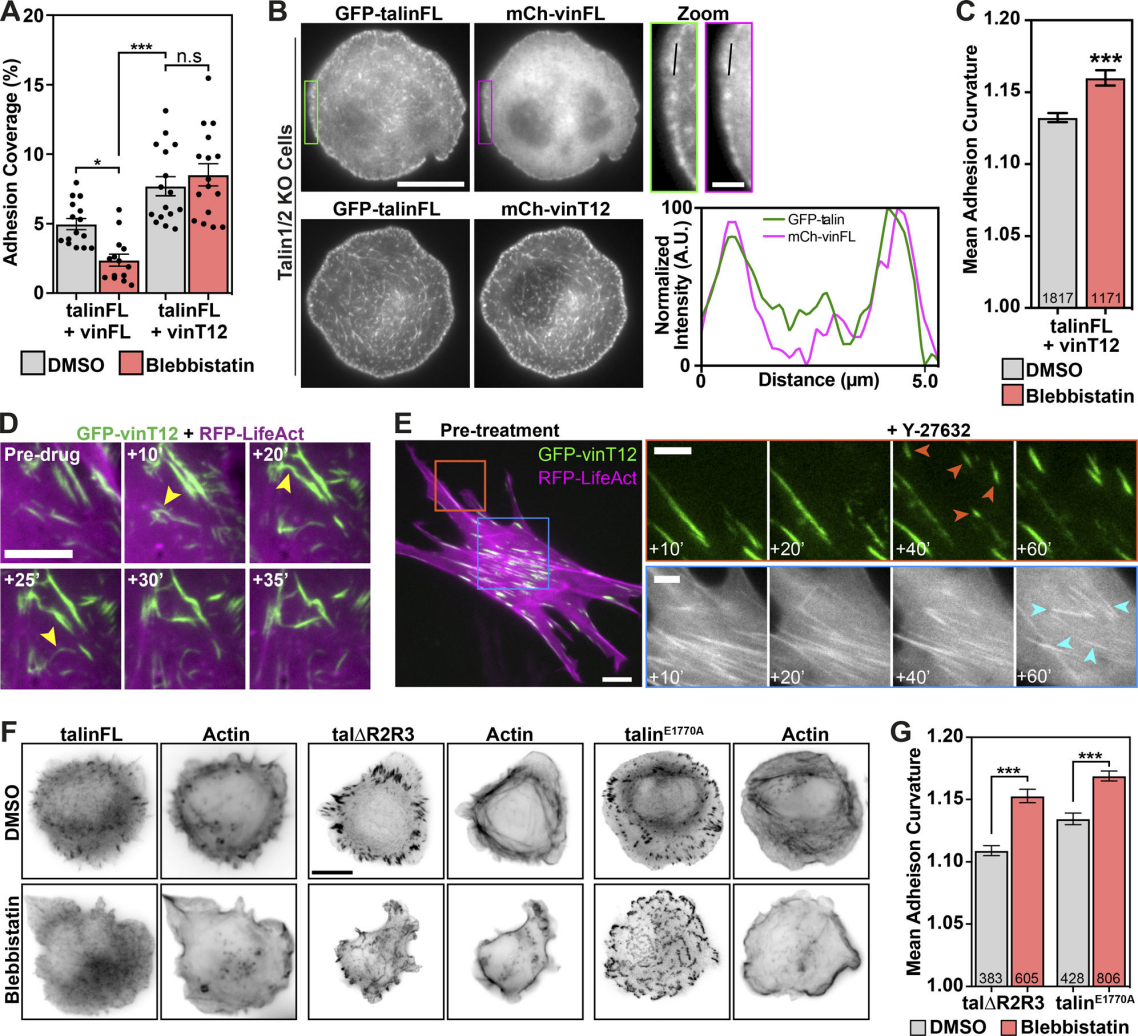
**Activated vinculin or talin support adhesion formation under tension-release conditions. (A)** TalinKO cells coexpressing GFP-talinFL with either autoinhibited mCh-vinFL or activated mCh-vinT12 were pretreated in suspension with blebbistatin (50 µM) or an equivalent volume of DMSO for 45 min. Cells were fixed after spreading on fibronectin-coated glass for 15 min. The percentage of the cell consisting of adhesions was quantified from the GFP signal. Graphs show the mean and SEM; *n* = 15 (talinFL + vinFL DMSO), 14 (talinFL + vinFL Blebbistatin), 16 (talinFL + vinT12 DMSO), and 16 (talinFL + vinT12 Blebbistatin) cells; results are representative of three independent experiments. *, P < 0.05; ***, P < 0.001; one-way ANOVA with Holm-Sidak’s multiple comparison test. **(B)** Representative images of talinKO cells as described above. Scale bar indicates 10 µm (scale bar in the magnified region indicates 2 µm). Line profile shows that mCh-vinFL and GFP-talinFL colocalize at peripheral adhesion structures, whereas mCh-vinT12 and GFP-talinFL colocalize at all adhesions throughout the cell. **(C)** Quantification of the curvature of talin-positive structures in these cells. Graphs show the mean and SEM; *n* = 1,817 structures analyzed from 16 (DMSO) and 1,171 structures analyzed from 15 (blebbistatin) cells. ***, P < 0.001, unpaired two-tailed *t* test. Results are representative of three independent experiments. **(D)** Still frame images from a video of a vinculinKO MEF expressing GFP-vinT12 (green) and RFP-LifeAct (magenta) before and during treatment with Y-27632 (50 µM). Note the buckling and bending of the adhesions (arrowheads). Scale bar indicates 5 µm. **(E)** VinculinKO MEF expressing GFP-vinT12 together with RFP-LifeAct treated with Y-27632 (50 µM) and imaged every minute for 60 min. Active vinculin supports the formation and growth of new adhesions (upper panels, orange arrowheads), with actin stress fibers remaining bundled at the center of the cell (lower panels, cyan arrowheads). Scale bar indicates 10 µm (scale bars in magnified regions indicate 5 µm). **(F)** TalinKO cells expressing GFP-talΔR2R3 or GFP-talin^E1770A^, after DMSO or blebbistatin treatment (as described above in A), with phalloidin staining shows GFP-positive structures form during cell spreading without intracellular tension. Scale bar indicates 10 µm. **(G)** Quantification of the curvature of talin-positive structures in these cells. Graph shows the mean and SEM; *n* = 383 (talΔR2R3 DMSO), 605 (talΔR2R3 blebbistatin), 428 (talin^E1770A^ DMSO), and 806 (talin^E1770A^ Blebbistatin) structures analyzed from 15 cells for each condition. ***, P < 0.001, one-way ANOVA with Holm-Sidak’s multiple comparison test. Results are representative of three independent experiments.

**Figure S4.**
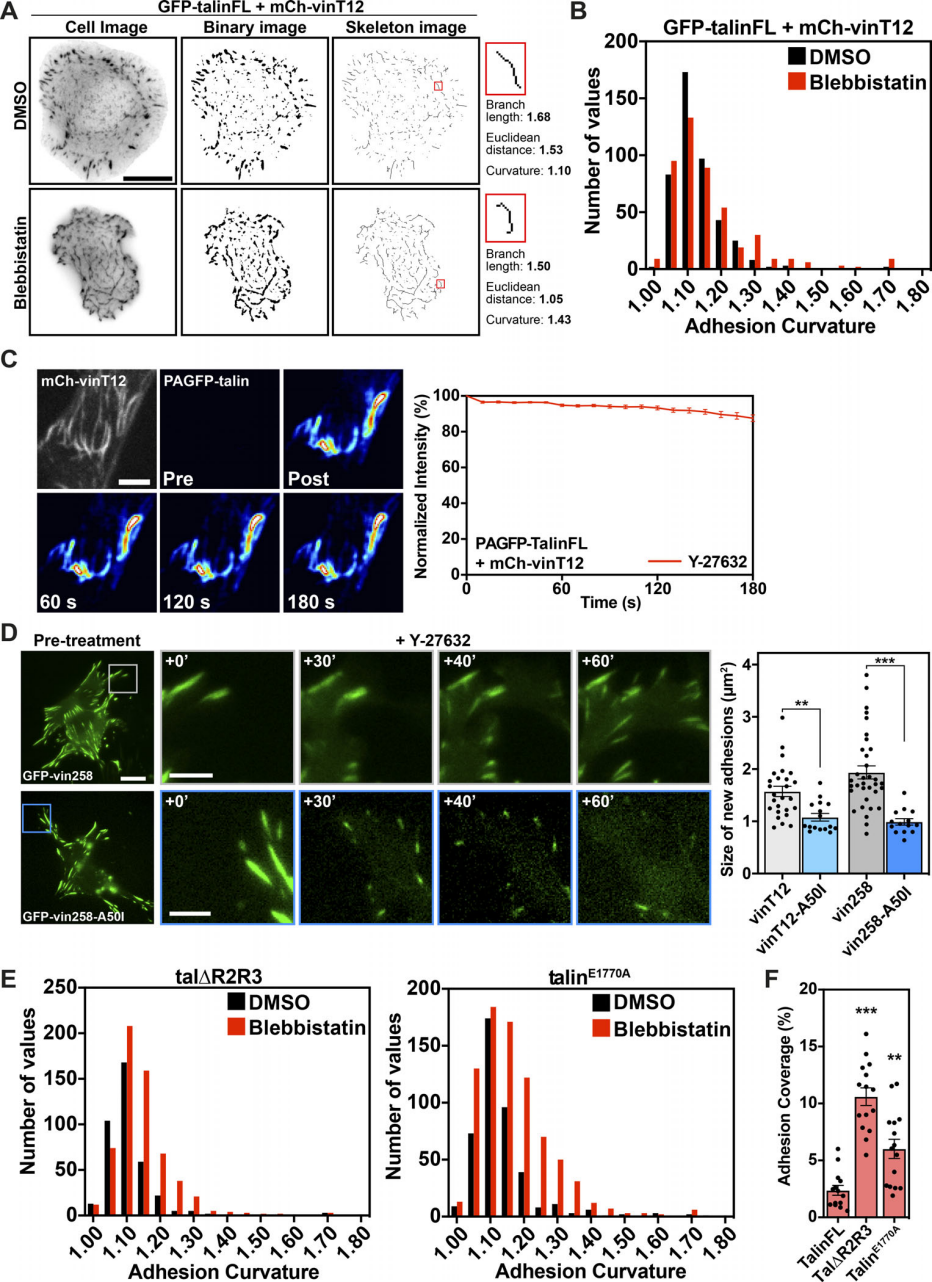
**Active talin constructs form disorganized adhesions when spreading in the absence of intracellular tension. (A)** Workflow of image processing for adhesion curvature calculation. The GFP-talin signal was background subtracted and thresholded to generate a binary image, which was then used to generate a skeleton image. The length and curvature of each branch >0.2 µm was used to calculate the curvature of the adhesion.** (B) **Histograms of adhesion curvature calculated from talinKO cells coexpressing GFP-talinFL and mCh-vinT12 treated in suspension with either DMSO or blebbistatin. **(C)** FLAP of PAGFP-talinFL in NIH3T3 cells coexpressing mCh-vinT12 after treatment with Y-27632 (50 µM) shows that the interaction between the two proteins is extremely stable (Mf < 95%) in the absence of intracellular tension. Scale bar indicates 5 µm. Error bars represent SEM; *n* = 30 measurements from five cells. Results are representative of three independent experiments. **(D)** Still-frame images from movies of vinculinKO MEFs expressing GFP-vin258 or GFP-vin258-A50I treated with Y-27632 (50 µM). Scale bar indicates 10 µm (scale bar in magnified regions indicates 5 µm). The growth and correct maturation of new adhesions requires the interaction between vinculin and talin. Error bars represent SEM; *n* = 26 (vinT12), 16 (vinT12-A50I), 34 (vin258), and 14 (vin258-A50I) adhesions; **, P < 0.01; ***, P < 0.001, one-way ANOVA with Holm-Sidak’s multiple comparisons test; results are representative of three independent experiments. **(E)** Histograms of adhesion curvature calculated from talinKO cells expressing GFP-tal∆R2R3 or GFP-talin^E1770A^, after DMSO or blebbistatin treatment as above. **(F)** Quantification of the percentage of the cell consisting of adhesions (quantified from the GFP signal) in talinKO cells expressing GFP-tal∆R2R3 or GFP-talin^E1770A^. Cells were pretreated in suspension with blebbistatin (50 µM) or an equivalent volume of DMSO, for 45 min, before being seeded onto fibronectin-coated glass and fixed after 15 min of spreading. Graphs show the mean and SEM; *n* = 14 (talinFL), 15 (tal∆R2R3), and 15 (talin^E1770A^) cells. Results are representative of three independent experiments; **, P < 0.01; ***, P < 0.001, significance against talinFL (one-way ANOVA with Holm-Sidak’s multiple comparisons test).

Given the surprising finding that talin and vinculin can interact at mitochondria in the absence of force, we sought to clarify the role of actomyosin-mediated tension during FA formation and maturation. Tension release inhibits maturation of adhesion complexes into streak-like FAs, although small adhesions complexes containing talin and vinculin still remain (Stutchbury et al., 2017). To mimic experiments at mitochondria, we coexpressed activated mCh-vinT12 together with autoinhibited GFP-talinFL in talin knockout (talinKO) cells and compared FA formation to cells coexpressing autoinhibited mCh-vinFL and GFP-talinFL. We created a force-free environment by pretreating cells with the tension-releasing drug blebbistatin (50 µM) for 45 min, before allowing them to spread on fibronectin for 15 min. Cells coexpressing mCh-vinFL and GFP-talinFL only formed small peripheral adhesions (Fig. 5 A). In contrast, cells coexpressing mCh-vinT12 and GFP-talinFL formed larger adhesions throughout the cell that were positive for both talin and constitutively active vinculin (mCh-vinT12; Fig. 5 B). However, although these adhesions were streak-like, as seen in mature FAs of nontreated cells, they were random in orientation and often bent (Fig. 5 C and Fig. S4, A and B).

We wondered whether this buckling seen in cells coexpressing mCh-vinT12 and GFP-talinFL was due to the lack of tension resulting from inhibition of the actomyosin machinery. To test this, we treated vinKO mouse embryonic fibroblasts (MEFs) expressing GFP-vinT12 and RFP-LifeAct with Y-27632 (50 µM). Remarkably, the previously well-organized and streak-like FAs with vinT12 attached to actin stress fibers started to bend and buckle without being disassembled (Fig. 5 D and Video 1). FLAP experiments revealed these associations to be extremely stable (Mf < 20%; Fig. S4 C). Interestingly, new adhesions formed at the cell periphery that seemingly grow even under tension-release conditions (Fig. 5 E and Video 1). We observed the same results when expressing a GFP-vin258 construct in vinKO MEFs (Fig. S4 D). Importantly, the growth and stabilization of new adhesions was dependent on the ability of vinculin to bind to talin, since expression of either a GFP-vinT12-A50I or a GFP-vin258-A50I construct in these cells significantly reduced the size of the newly forming adhesions (Fig. S4 D). These experiments suggest that in FAs, activated vinculin can bind talin independently of tension and link the complex to actin filaments.

**Video 1.** **Active vinculin (vinT12) supports adhesion formation under tension-release conditions.** Time-lapse confocal microscopy movie of a vinculinKO MEF cell coexpressing GFP-vinculinT12 and RFP-LifeAct. Images were acquired every minute for 1 h, starting 10 min after the addition of Y-27632 (50 µM). Scale bar indicates 10 µm. Blue insert shows the formation and growth of new adhesions and stress fibres. Yellow insert shows the buckling and bending of existing adhesions.

We next investigated whether active talin constructs could support adhesion maturation in the absence of forces (after treatment in suspension with blebbistatin as described above). As for vinT12, expressing either talΔR2R3 or talin^E1770A^ in talinKO cells promoted the formation of talin-positive elongated and disorganized FA structures that were larger than those found in control cells expressing talinFL (Fig. 5, F and G; and Fig. S4, E and F).

Taken together, while there seems to be some bundling activity through the binding of talin and vinculin, full maturation into a stress-fiber–associated tensile FA requires actomyosin-mediated tension.

### Paxillin can bind to both talin and vinculin independently of force

**Figure 6.**
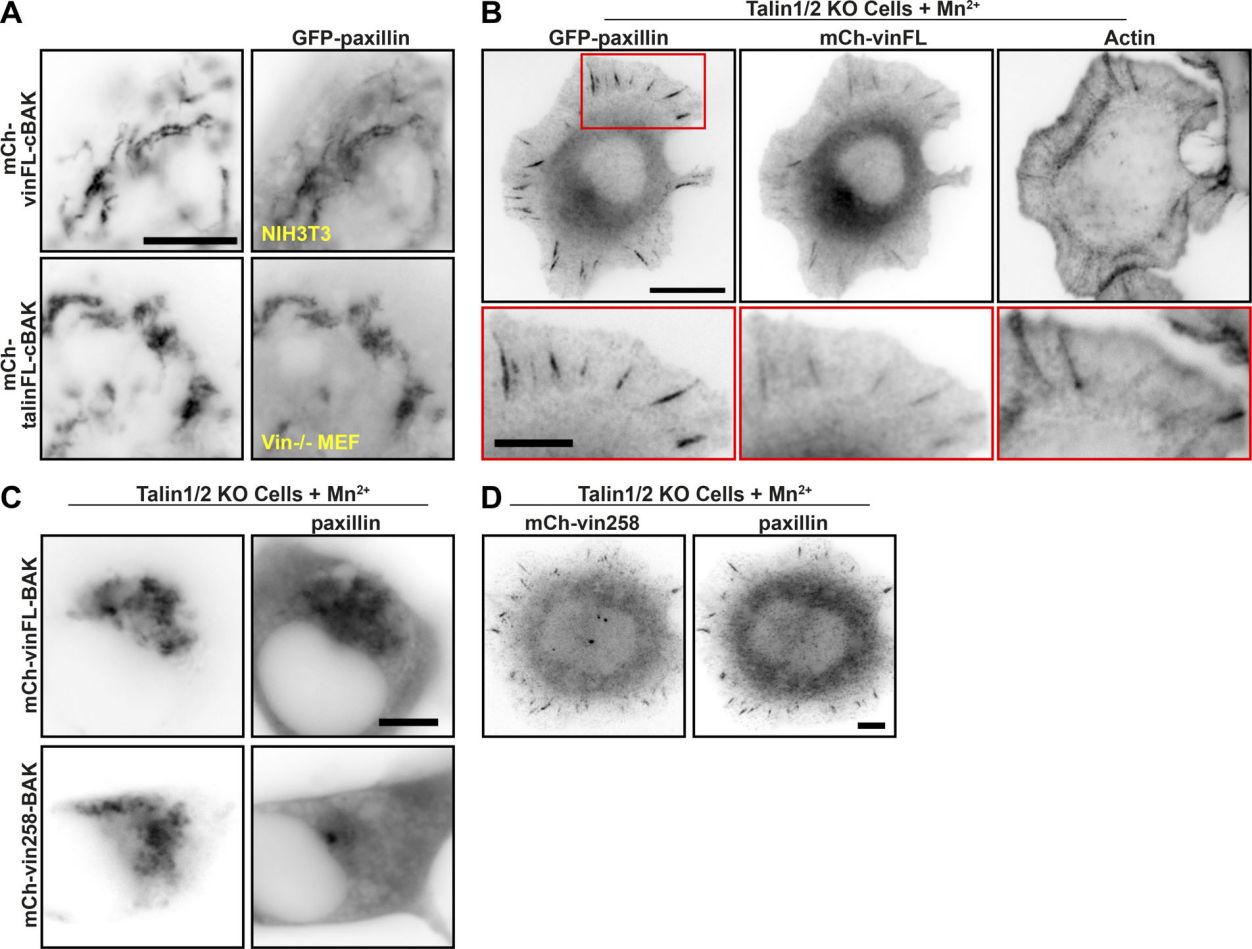
**Paxillin can bind both talin and vinculin when either is in an inactive conformation. (A)** Coexpression of GFP-paxillin with either mCh-vinFL-cBAK or mCh-talinFL-cBAK in NIH3T3 cells. The colocalization suggests that paxillin can bind to inactive vinculin or talin independently of forces. **(B)** TalinKO cells expressing GFP-paxillin and mCh-vinFL fixed and stained with 647-Phalloidin after Mn^2+^ (5 mM)-induced spreading on fibronectin. Note the colocalization of both in FAs. Scale bar in magnified region indicates 5 µm. **(C)** TalinKO cells expressing GFP-paxillin and mCh-vinFL-cBAK or mCh-vin258-cBAK spread on fibronectin in the presence of Mn^2+^ (5 mM). Note that mCh-vin258-cBAK does not recruit paxillin. **(D)** TalinKO cells expressing mCh-vin258-cBAK spread on fibronectin in the presence of Mn^2+^ (5 mM) were fixed after 1 h of spreading and stained for paxillin. Note the localization of mCh-vin258 to adhesions in these cells. Scale bars in A–D indicate 10 µm.

**Figure S5.**
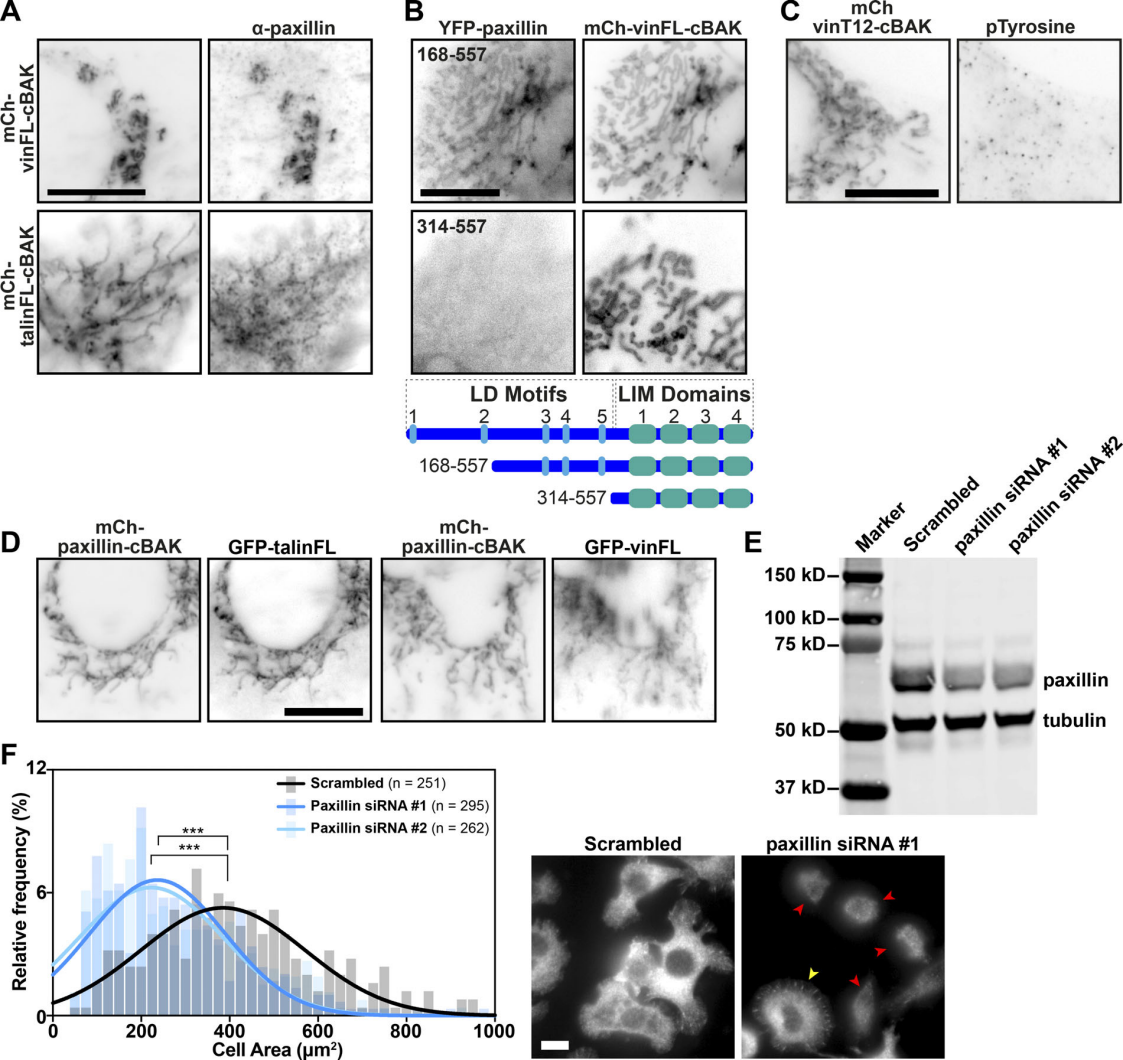
**Paxillin can recruit inactive vinculin and talin independently of force. (A)** Staining for endogenous paxillin in NIH3T3 cells expressing either mCh-vinFL-cBAK or mCh-talinFL-cBAK shows paxillin is recruited to both inactive vinFL-cBAK and talinFL-cBAK. **(B)** Colocalization of YFP-paxillin168-557 (upper panel) or YFP-paxillin314-557 (lower panel) with mCh-vinFL-cBAK shows a stretch of paxillin containing the third, fourth, and fifth LD motifs are required for the interaction between paxillin and mCh-vinFL-cBAK. **(C)** Staining for phosphotyrosine in NIH3T3 cells expressing mCh-vinT12-cBAK reveals that no tyrosine phosphorylation is present at these mitochondria. **(D)** Coexpression of mCh-paxillin-cBAK with either GFP-vinFL or GFP-talinFL in NIH3T3 cells shows mCh-paxillin-cBAK is able to recruit either GFP-vinFL or GFP-talinFL to mitochondria. Scale bars in A–D indicate 10 µm. **(E)** Cell lysates from talinKO cells transfected with siRNA targeting paxillin were separated by SDS-PAGE and stained for paxillin and tubulin. Either siRNA sequence reduced paxillin levels by ∼60%. **(F)** TalinKO cells transfected with two separate siRNA sequences targeting paxillin were plated on fibronectin in the presence of Mn^2+^ (5 mM) and fixed after 1 h. Cell area was measured from 10 images (*n* = 251 [Scrambled], 295 [Paxillin siRNA #1], and 262 [Paxillin siRNA #2] cells) and is presented as a histogram showing the percentage of cells with different areas with bins of 25 µm. Results are representative of three independent experiments; ***, P < 0.001 (one-way ANOVA with Dunnett’s multiple comparison test). Note that paxillin knockdown strongly reduces the number of spread cells; cells still containing residual paxillin are able to spread (yellow arrowhead), unlike those lacking paxillin (red arrowheads). Scale bar indicates 20 µm.

Talin and vinculin orchestrate adhesion signaling by binding to many other proteins within the FA, including paxillin, which binds to both talin and vinculin (Turner et al., 1990; Wood et al., 1994; Zacharchenko et al., 2016). Additionally, paxillin is thought to contribute to the recruitment of vinculin to focal complexes (Case et al., 2015; Pasapera et al., 2010). To examine whether forces are required for the association of paxillin with talin and vinculin, we probed their interaction using the mitochondrial targeting assay. Interestingly, GFP-paxillin and endogenous paxillin were recruited to both vinFL-cBAK and talinFL-BAK (Figs. 6 A and S5 A). The paxillin–vinculin interaction at mitochondrial was dependent on the presence of regions in paxillin that contained the leucine-aspartate repeat motifs 3–5 (Fig. S5 B) and occurred independent of tyrosine phosphorylation, which was not detected at mitochondria (Fig. S5 C). Since the wild-type forms of talin and vinculin do not interact with each other at mitochondria, we conclude that they can both associate with paxillin in their inactive form. Similarly, a mCh-paxillin-cBAK construct was able to recruit both GFP-talin and GFP-vinculin to mitochondria (Fig. S5 D).

To examine the potential role of paxillin in recruiting vinculin to adhesions in the absence of talin, we conducted experiments in talinKO cells. Under normal conditions, these cells neither adhere nor spread, but activation of integrins by Mn^2+^ induces spreading on fibronectin (Theodosiou et al., 2016). Surprisingly, in Mn^2+^-stimulated talinKO cells, we found that both paxillin and vinculin localized to FA-like structures located along filopodia-like actin bundles embedded in protruding lamellipodia (Fig. 6 B).

From these results, we hypothesized that paxillin may be involved in recruiting vinculin to these adhesions structures. To test this, we initially performed siRNA-mediated knockdown of paxillin (Fig. S5 E), but these cells were impaired in cell spreading and adhesion formation (Fig. S5 F), thus confirming other reports that paxillin mediates cells spreading (Hagel et al., 2002; Pasapera et al., 2010; Theodosiou et al., 2016; Wade et al., 2002).

To determine in more detail whether paxillin is involved directly in vinculin recruitment, we examined the localization of vin258 comprising the D1 domain of the vinculin head. This construct, lacking the reported paxillin-binding site in the vinculin tail region (Turner et al., 1990; Wood et al., 1994), when fused to BAK did not recruit paxillin (Fig. 6 C), but GFP-vin258 without BAK readily localized to adhesions of talinKO cells treated with Mn^2+^ (Fig. 6 D). Together, these results suggest that there are other proteins besides paxillin and talin that can mediate vinculin recruitment to FAs.

### The talin–vinculin interaction is essential for clutch engagement and force transduction

**Figure 7.**
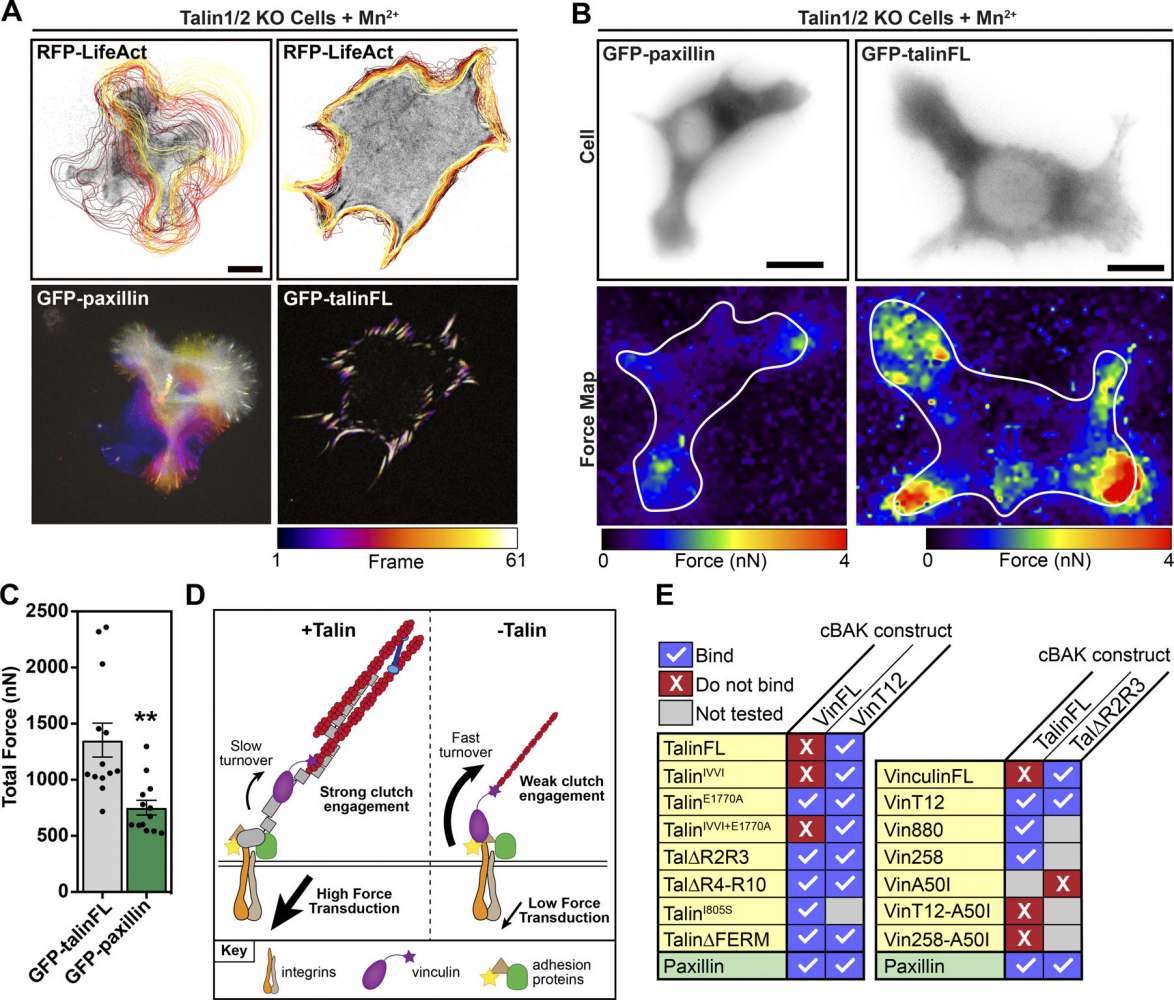
**The talin–vinculin interaction is required for efficient force transduction. (A)** Live-cell imaging of talinKO cells coexpressing either GFP-paxillin or GFP-talinFL with RFP-LifeAct (top panel). The cell edge was traced over time using the RFP-LifeAct signal. Black outline indicates the cell position at the first frame; yellow indicates the position in the last frame. Temporal color maps of adhesion movement obtained from the GFP signal of the same cell (lower panel) show that cells without talin after Mn^2+^ treatment are highly dynamic compared with talin-expressing Mn^2+^-treated cells. Images were acquired every 2 min for 2 h. **(B)** Representative force maps of talinKO cells expressing either GFP-paxillin or GFP-talinFL spread on a PAA hydrogel containing fluorescent beads for traction force microscopy. White line indicates the outline of the cell. Blue color indicates regions of low force exertion; red indicates regions of high force exertion. Scale bars in A–D indicate 10 µm. **(C)** Quantification of the total force exerted per cell from traction force microscopy experiments. Graphs show the mean and SEM; *n* = 13 cells, results are representative of three independent experiments. **, P < 0.01, unpaired two-tailed *t* test. **(D)** Schematic showing the role of talin in force transduction. In wild-type cells (+talin), vinculin (purple) reinforces the link between talin (gray) and actin, engaging the molecular clutch for efficient force transduction, stabilizing adhesion turnover. Without talin (−talin), vinculin is weakly anchored to additional adhesion proteins and is only able to transduce low forces. These adhesions are unstable and rapidly turned over. **(E)** Tables summarizing the binding between indicated constructs.

Vinculin is an integral component of the molecular clutch; its engagement with the retrograde flow of F-actin at the leading edge generates traction forces (Thievessen et al., 2013). We next investigated whether vinculin alone at the Mn^2+^-induced adhesions of talinKO is sufficient for clutch engagement and force transduction. Live-cell imaging of talinKO cells expressing GFP-paxillin and RFP-LifeAct stimulated with Mn^2+^ revealed that the actin cytoskeleton of these cells was extremely dynamic and that the adhesions had a fast turnover. This was in stark contrast to talinKO cells expressing GFP-talinFL and RFP-LifeAct stimulated with Mn^2+^, which displayed very stable adhesions (Fig. 7 A and Video 2). Furthermore, traction force microscopy in the presence of Mn^2+^ revealed that FAs in talinKO cells generated only ∼50% of traction forces compared with control cells expressing GFP-talinFL (Fig. 7, B and C). Overall, these experiments demonstrate that the interaction between talin and vinculin is essential for engagement of the molecular clutch for control of mechanotransduction from the cytoskeleton to the ECM (Fig. 7 D).

**Video 2.** **Integrin activation in talinKO cells supports adhesion formation at sites of actin bundles.** Time-lapse confocal microscopy movies of talinKO cells coexpressing GFP-paxillin (upper left panel) and RFP-LifeAct (upper central panel), or coexpressing GFP-talinFL (lower left panel) and RFP-LifeAct (lower central panel). In both cases, cells were stimulated with Mn^2+^ (5 mM) for 1 h before imaging. Without talin (upper panels), cells have an extremely dynamic actin cytoskeleton and adhesions are rapidly turned over. Talin expression (lower panels) acts to stabilize adhesions and the actin cytoskeleton. Images were acquired every 2 min for 2 h. Images have been corrected for bleaching and smoothened. Scale bar indicates 10 µm.

## Discussion

The development of FAs is a highly dynamic process where growth is associated with increasing molecular complexity and actomyosin contractility. The short lifetime of nascent adhesions makes studying mechanisms governing the interactions between proteins within these structures difficult. Additional problems arise due to the complex nature of protein–protein interactions in mature FAs. To overcome these barriers, we used a mitochondrial-targeting assay to examine molecular interactions at mitochondria, which move in a force-free environment in the cell’s cytoplasm independent of actomyosin. We modified the assay used previously (Bubeck et al., 1997; Cohen et al., 2006; Maartens et al., 2016) by using cBAK instead of ActA as the targeting motif. In contrast to ActA, the cBAK motif stably integrates into the outer mitochondrial membrane (Fig. S1 C; Schellenberg et al., 2013), allowing the assessment of protein–protein binding strength using FLAP and nullifying movement of proteins between FAs and mitochondria. Our results provide important new insights into the molecular mechanisms underpinning the talin–vinculin interaction and the assembly of FAs.

In vitro stretching experiments show that force exerted on the helical bundles of the talin rod exposes the multiple cryptic VBSs contained therein (del Rio et al., 2009; Yao et al., 2014, 2016), suggesting force across talin is likely to be required for the vinculin–talin interaction. However, the results from our force-free mitochondrial targeting system do not fit this model, since active vinculin stably interacted with talinFL (Figs. 2 A and S1 B), and the unstretched talin rod or selected deletion mutants interacted with vinFL (Figs. 3 and S3 A). Moreover, none of the above interactions were affected by blebbistatin, Y-27632, or cytochalasin D, demonstrating that force exerted by actomyosin contraction is not essential for activated vinculin to bind talinFL (Fig. 2 B). Similar stable interactions between activated vinculin constructs and full-length talin have been observed in the cytoplasm of *Drosophila melanogaster* embryos (Maartens et al., 2016), a site where forces would not be expected to contribute to complex assembly.

How then can one explain the discrepancy between “force-induced” models and our data, as well as the previously reported interaction between vinculin D1 (vinD1) and talin fragments containing R3 in the absence of force (Goult et al., 2013b)? Careful inspection of in vitro single-molecule experiments show that there is some residual binding of the vinD1 domain to unstretched talin rod domains (del Rio et al., 2009; Goult et al., 2013b). Moreover, the detection of a 5-pN transition corresponding to R3 unfolding in the presence of vinD1 may be rather uncertain due to the significant noise level (Yao et al., 2016). Temperature could be an additional factor contributing to the outcome of the experiments; in vitro stretching experiments were conducted at room temperature (Yao et al., 2014), whereas the cellular milieu is 37°C, which facilitates talin unfolding and vinculin binding (Goult et al., 2013b; Patel et al., 2006). Perhaps the temperature of the single-molecule stretching experiments (Yao et al., 2014) was sufficiently low to stabilize R3 and inhibit interaction with vinculin. However, to reveal whether forces are required for talin–vinculin interactions in more stable helical bundles in the rod domain will require performing stretch experiments at physiological temperatures.

While inactive vinFL-cBAK does not bind inactive talin, relief of talin autoinhibition through a single point mutation in R9 (E1770A) or deletion of the FERM domain (talinΔFERM) is sufficient to induce binding (Figs. 4 B and S3 A). The interactions of the rod-deletion constructs talΔR2R3 or talΔR4-R10 with vinFL-cBAK (Fig. 3 C) suggest that there are at least two rod domains that can interact with vinculin in the absence of force. One of them is R3, which appears to be a critical determinant of the vinculin–talin interaction, since stabilization of this domain with the IVVI point mutations (Goult et al., 2013b) prevents the interaction with the E1770A-activated talin (Fig. 4 E). At the same time, point mutations I805S and L897S that destabilize R3 (Rahikainen et al., 2017) enable binding of talinFL to vinFL-cBAK, without the need for mutations that relieve autoinhibition (Figs. 4 D and S3 B), support the idea that talin autoinhibition and the stability of R3 go hand in hand. While this manuscript was in revision, Dedden et al. (2019) found that one VBS on talin is available after the release of talin autoinhibition. This outcome, from experiments using cryo-EM to study the talin structure in vitro, is in line with our results from experiments conducted in cells.

Do the force-independent interactions between talin and vinculin we observe exclude a role for forces in the activation process and complex formation? To engage force, talin needs to interact simultaneously with integrin and actin. The F3 integrin-binding site in the autoinhibited talin is blocked by the interaction with the R9 domain, and the actin-binding sites in the rod are inaccessible, as the cytosolic talin does not interact with actin. Integrin binding may be promoted by the interaction with the phosphatidylinositol 4,5-bisphosphate (PIP_2_)–enriched membrane, which facilitates displacement of R9 from F3 (Song et al., 2012). Our data demonstrate that the relief of this autoinhibition will allow vinculin binding to R3 that would be expected to potentiate subsequent actin bundling (Atherton et al., 2015). In a complementary mechanism, independent vinculin activation promotes vinculin binding to autoinhibited talin and its subsequent activation.

Once talin is activated and bound to integrins, increased actin binding, supported by actomyosin forces, would be expected to potentiate subsequent actin bundling (Atherton et al., 2015). All these factors are likely to further increase lifetimes of an open, activated talin configuration. This is in line with previous observations demonstrating a role for actin-binding site ABS2 in the stability of talin in FAs, the availability of which is regulated by active vinculin binding to talin (Atherton et al., 2015). Similar to talin, vinculin conformation is reportedly modulated by PIP_2_ (Chinthalapudi et al., 2014; Gilmore and Burridge, 1996; Johnson et al., 1998) and actin binding/bundling and possibly force may shift the equilibrium toward a more open activated state (Chen et al., 2006), similar to the one reported by collision-induced unfolding experiments (Chorev et al., 2018). This in turn will stabilize the dynamics of talin and vinculin, reducing adhesion turnover (Rothenberg et al., 2018).

Critical to such a mechanism is the recruitment of talin and vinculin to the adhesion sites. Several proteins have already been shown to mediate this process, including Rap1 and RIAM for talin (Gingras et al., 2019; Goult et al., 2013b; Lafuente et al., 2004) or VASP for vinculin (Hüttelmaier et al., 1998). Our results highlight the potential role of paxillin in the recruitment process and suggest that it may couple talin and vinculin localization through the interaction with both proteins in their inactive states (Fig. 6 A). This is in line with reports that show that paxillin promotes the recruitment of vinculin in an inactive conformation to the integrin signaling layer of FA (Case et al., 2015) and that paxillin can associate with vinculin in the cytoplasm (Hoffmann et al., 2014). Such a cytoplasmic paxillin–vinculin complex could be driven to nascent adhesions by the interaction between paxillin and kindlin2 (Böttcher et al., 2017), which was reported to be critical for cell spreading (Theodosiou et al., 2016). However, while paxillin may be able to contribute to vinculin recruitment in adhesions, our observation of the isolated vinculin D1 domain (vin258) localizing to Mn^2+^-induced adhesions in talin-deficient cells (Fig. 6 D) suggests other binding partners to this domain participate in this process.

**Figure 8.**
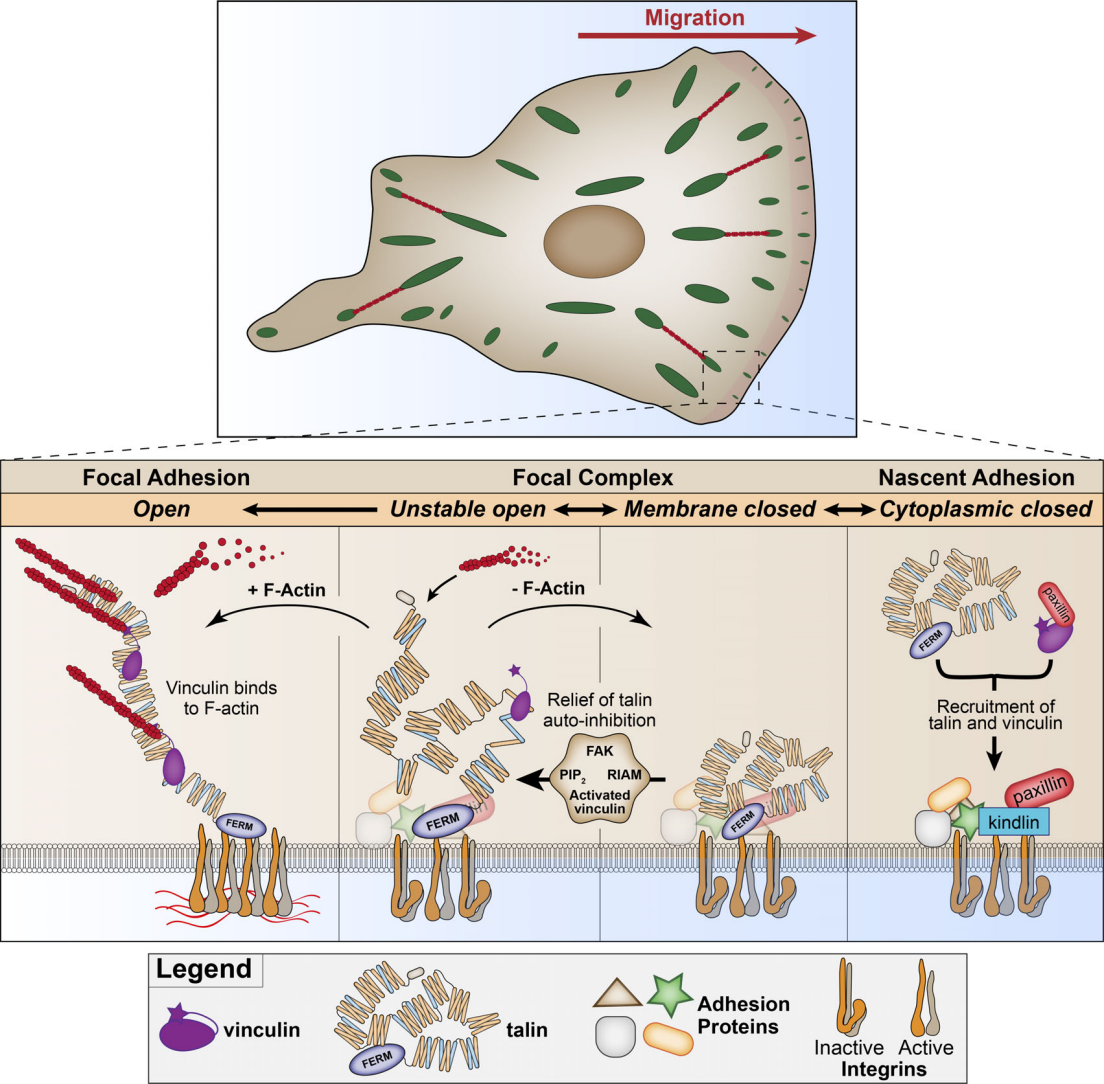
**Model of early events in adhesion formation during polarized migration.** See the last paragraph of the Discussion.

Based on our results and the previous literature, we propose the following model for talin recruitment, activation, and vinculin binding during polarized cell migration (Fig. 8). At the leading edge, actin polymerization driven by Rap1 and lamellipodin/RIAM (Lagarrigue et al., 2015) and the actin-binding proteins Arp2/3 and VASP (Lafuente et al., 2004) position adhesion proteins, including FAK (Serrels et al., 2007; Swaminathan et al., 2016), kindlin, and paxillin (Böttcher et al., 2017; Theodosiou et al., 2016), to form preadhesion complexes. These adhesion proteins can recruit talin (Lawson et al., 2012) and vinculin from the cytoplasm to the membrane. Relief of talin autoinhibition through biochemical factors (e.g., PIP_2_) changes talin conformation to an unstable open state that has a short lifetime and a tendency to refold. Inactive vinculin is able to bind to talin in this configuration (Fig. 4) and become activated (Izard et al., 2004). Subsequent F-actin binding (from the retrograde flow at the leading edge) and forces applied to the C-terminal ABS3 of talin, and the exposed F-actin–binding site in the vinculin tail and talin ABS2, will stabilize the complex. Actomyosin-mediated tension can then direct adhesion growth and maturation (Fig. 5), required for efficient force transmission and mechanosensing (Fig. 7).

## Materials and methods

### Cell culture

NIH3T3s and vinculin^−/−^ MEFs were cultured in DMEM supplemented with 10% FCS (Lonza), 1% L-glutamine (Sigma), and 1% nonessential amino acids (Sigma). Talin1 and talin2 double-null cells (Atherton et al., 2015) were cultured in DMEM:F12 (Lonza) supplemented with 10% FCS, 1% L-glutamine, 15 µM Hepes (Sigma), and 1% nonessential amino acids.

Transient transfections were performed using Lipofectamine and Lipofectamine Plus reagents (Invitrogen), as per the manufacturer’s instructions. For live-cell imaging and fixed-cell imaging, cells were cultured on glass-bottom dishes (IBL) coated with bovine fibronectin (Sigma) at a final concentration of 10 µg ml^−1^.

### Generation of cBAK-tagged constructs

To generate vinculin-cBAK constructs, assembly PCR was first used to generate the 108-bp mitochondrial targeting sequence from cBAK. The following four primers were used: (1) forward external, 5′-TAT​GAA​TTC​TTG​CGT​AGA​GAC​CCC​ATC​CTG-3′, (2) forward internal, 5′-CCC​ATC​CTG​ACC​GTA​ATG​GTG​ATT​TTT​GGT-3′, (3) reverse internal, 5′-ATC​TGT​GTA​CCA​CGA​ATT​GGC​CCA​ACA​GAA-3′, and (4) reverse external, 5′-TAT​GGT​ACC​TCA​TGA​TCT​GAA​GAA​TCT​GTG-3′. The 5′ and 3′ end primers (external) contained EcoRI and KpnI restriction digestion sites, respectively.

Site-directed mutagenesis was used to remove the stop codon from vinculin constructs and add an EcoRI restriction site. Digestion with EcoRI and KpnI FastDigest enzymes (Fermentas) was used to clone the cBAK fragment into the vinculin (*Gallus gallus*) constructs in Clontech C1 vectors.

To generate talin-cBAK constructs, a 1,133-bp sequence was synthesized (Genewiz), consisting of 1,031 bp from talin1 (*Mus musculus*) joined to the cBAK fragment (5′-TTG​CGT​AGA​GAC​CCC​ATC​CTG​ACC​GTA​ATG​GTG​ATT​TTT​GGT​GTG​GTT​CTG​TTG​GGC​CAA​TTC​GTG​GTA​CAC​AGA​TTC​TTC​AGA​TCA​TGA-3′) with the talin stop codon removed, flanked by a 5′ SalI restriction site and a 3′ SacII restriction site. This fragment was cloned into the GFP- and mCherry-talin constructs in Clontech C1 vectors by restriction digest, using the SalI restriction site located in the talin1 gene and the SacII restriction site present in the Clontech C1 vector.

### Antibodies and reagents

Samples were fixed in 4% PFA, warmed to 37°C, for 15 min before being washed thrice with PBS. For immunofluorescence, samples were permeabilized at room temperature with Triton X-100 (0.5%) for 5 min before being washed thrice. The following primary antibodies were used at the indicated dilutions (in 1% BSA): mouse anti-paxillin (clone 349, 610051, 1:400; BD Transduction Labs), mouse anti-vinculin (hVin1, V9131, 1:400; Sigma), rat anti-β1 integrin (9EG7, 553715, 1:200; BD Biosciences), and mouse anti-phosphotyrosine (4G10, 05-321, 1:400; Merck). Actin was visualized using Texas red–conjugated Phalloidin (Thermo Fisher), diluted 1:400. Secondary antibodies (Dylight 488– or 594–conjugated donkey anti-mouse or anti-rabbit) were purchased from Jackson ImmunoResearch and used at a dilution of 1:500.

Y-27632 (Tocris Bioscience) was diluted in dH_2_0 and used at a final concentration of 50 µM. Blebbistatin (Tocris Bioscience) and cytochalasin D (Sigma) were diluted in DMSO (Sigma) and used at a final concentration of 50 µM and 25 µg ml^−1^, respectively. MitoTracker Deep Red FM (Thermo Fisher) was dissolved in DMSO to a concentration of 1 mM. Prior to use, the stock was diluted in prewarmed medium at a final concentration of 200 nM before being added directly to cells 30 min before imaging.

Mutagenesis was performed using the QuikChange Lightning site-directed mutagenesis kit (Agilent) according to the manufacturer’s instructions.

### Microscopy

FLAP experiments were performed as described previously (Stutchbury et al., 2017). Images were acquired using a CSU-X1 spinning disc confocal (Yokagowa) on a Zeiss Axio-Observer Z1 microscope with a 60×/1.40 Plan-Apochromat objective (Zeiss), Evolve EMCCD camera (Photometrics), and motorized XYZ stage (ASI). The 405-, 488-, and 561-nm lasers were controlled using an acousto-optic tunable filter through the laserstack (Intelligent Imaging Innovations; 3I) allowing both rapid “shuttering” of the laser and attenuation of the laser power. One hour before imaging the medium was changed to prewarmed Ham’s F-12 medium supplemented with 25 mM Hepes buffer, 1% FCS, 1% penicillin/streptomycin, and 1% L-glutamine. Slidebook software (3I) was used to capture images every 10 s for 5 min. Temperature throughout imaging was maintained at 37°C. Movies were analyzed using ImageJ; the intensities of the postactivated PAGFP at mitochondria were measured manually using ImageJ. Values were normalized to the intensity of the first postactivation image. Graphs were prepared using Prism 8 (GraphPad).

Images of fixed samples in PBS were acquired at room temperature using a Zeiss AxioObserver Z1 wide-field microscope equipped with a 100×/1.4-NA oil objective and an Axiocam MRm camera, controlled by Zeiss Axiovision software. Samples were illuminated using a mercury bulb; specific band-pass filter sets were used to prevent bleed through from one channel to the next (for GFP, 38HE [Zeiss]; for mCherry, 43HE [Zeiss]).

### Live-cell imaging

Images of talinKO cells expressing GFP-paxillin or GFP-talinFL with RFP-LifeAct were acquired on a spinning-disk confocal microscope (CSU-X1; Yokagowa) supplied by Intelligent Imaging Innovations (3I) equipped with a motorized XYZ stage (ASI) maintained at 37°C, using a 100×/1.45 Plan-Apochromat oil objective (Zeiss) and an Evolve EMCCD camera (Photometrics). 1 h before imaging, the medium was changed to prewarmed Ham’s F-12 medium supplemented with 25 mM Hepes buffer, 1% FCS, 1% penicillin/streptomycin, and 1% L-glutamine, with 5 mM Mn^2+^ added as appropriate. The 488- and 561-nm lasers were controlled using an acousto-optic tunable filter through the laserstack. Cell edge tracing was performed with the QuimP plugins for FIJI (Baniukiewicz et al., 2018) using the signal from the RFP-LifeAct channel.

### Adhesion curvature quantification

Images of fixed samples in PBS were acquired using an Olympus IX83 inverted microscope equipped with a 60×/1.42 Plan-Apochromat oil objective (Olympus) using green and red Lumencor LED excitation and the Sedat filter set (Chroma 89000). Images were collected at room temperature using a Retiga R6 camera (Q-Imaging) controlled by Metamorph software. Adhesion curvature was calculated by first generating a binary image of the adhesions, which was then skeletonized. The Analyze Skeleton plugin for FIJI was used to extract the number of pixels and Euclidean distance for each line. Adhesion curvature was quantified for lines above 0.2 µm by dividing the total length by the Euclidean distance.

### Paxillin knockdown

TalinKO cells were transfected in a 6-well culture plate with either one of two siRNA sequences (Sigma) targeting mouse paxillin (paxillin siRNA 1, 5′-GUC​GUA​AAG​AUU​ACU​UCG​A-3′; paxillin siRNA 2, 5′-CAC​UUU​GUG​UGC​ACC​CAC​U-3′) using Lipofectamine 2000 as per the manufacturers’ instructions. After 48 h, one third of the cells were seeded onto fibronectin-coated glass in the presence of 5 mM Mn^2+^ and fixed 1 h after spreading. Cells were stained for paxillin (rabbit anti-paxillin, GTX125891; GeneTex) and imaged using the Zeiss AxioObserver Z1 wide-field microscope system, as described above, using a 40×/1.3 NA oil objective. Cell area was quantified manually using FIJI.

The remaining cells were lysed using RIPA buffer. 30 µg of protein was applied to SDS-PAGE, and proteins were transferred onto nitrocellulose membranes (Whatman). Membranes were blocked using casein blocking buffer (Sigma) and probed using primary antibodies diluted (1:1,000) in casein blocking buffer. Membranes were washed with Tris-buffered saline (10 mM Tris-HCl, pH 7.4, and 150 mM NaCl) containing 0.05% (vol/vol) Tween 20, followed by incubation with species-specific fluorescent dye–conjugated secondary antibodies (LI-COR Biosciences) diluted in PBS (1:5,000). Membranes were washed again and fluorescent signals were detected using the Odyssey infrared imaging system (LI-COR Biosciences).

### Traction force microscopy

Traction forces were quantified by preparing fibronectin coated polyacrylamide (PAA) hydrogels containing 0.2-µm-diameter red fluorescent beads (diluted 1:100, FluoSpheres carboxylate-modified red [580/605]; Molecular Probes) as described previously (Atherton et al., 2015). Briefly, PAA gels of 8 kPa were prepared by diluting 30% acrylamide/bis-acrylamide Protogel (EC-890; National Diagnostics) in PBS to 6%. The diluted PAA was degassed for 10 min before being polymerized using ammonium persulfate (A3678; Sigma) and tetramethylethylenediamine (T9281; Sigma). A thin layer (10 µl) of the PAA mixture was spread on a glass-bottom dish (D29-20-1-N; IBL) that had previously been cleaned with NaOH (0.1 M), functionalized by 3-aminopropyl trimethoxysilane (A3648; Sigma), and cross-linked with 0.5% gluteraldehyde (G5882; Sigma), by inverting a fibronectin-coated coverslip (50 µg/ml diluted in PBS) onto the droplet. The coverslip was carefully removed after 1 h, and the resulting traction force microscopy gels were washed thrice with PBS. TalinKO cells expressing either GFP-talinFL or GFP-paxillin were allowed to spread on the hydrogels in the presence of 5 mM Mn^2+^ for 1 h. Images were acquired of the cells and the beads under strain using an Olympus IX83 inverted microscope with a heated stage maintained at 37°C, with a 100× UPlanFL 100×/0.17 objective, using green and red Lumencor LED excitation and a Sedat filter set (Chrome 89000). Images were collected using a Retiga R6 camera (Q-Imaging). Cells were detached by adding 1% Triton X-100 for 45 min before images of the beads without strain were acquired. After aligning the stressed and relaxed bead images to correct for drift, the deformation of the hydrogel was calculated using particle image velocimetry plugins for ImageJ (Tseng et al., 2012). The total force was calculated by measuring the integrated density of the magnitude maps, using the whole cell area as a mask.

### Graphs and statistical analysis

All graphs were made using Prism 8 (GraphPad). Statistical analyses were performed using Prism 8 (GraphPad). Where appropriate, statistical significance between two individual groups was tested using a (two-tailed) *t* test. To test for significance between two or more groups, a one-way ANOVA was used with a Holm-Sidak’s multiple comparison test with a single pooled variance. Data distribution was assumed to be normal, but this was not formally tested.

### Online supplemental material

Fig. S1 shows that talinFL is recruited to truncated vinculin constructs at mitochondria. Fig. S2 shows that the interactions between active vinculin and talin, and active talin and vinculin, are through the canonical talin-binding site within the vinculin head. Fig. S3 shows that wild-type vinculin at mitochondria can bind to the talin rod, or to a talin construct with mutations in the R3 domain that increase the lability of the helical bundles. Fig. S4 shows that intracellular tension is required to direct the organization of adhesions formed by active talin constructs. Fig. S5 shows that paxillin can recruit both talin and vinculin in their inactive forms, independently of force. Video 1 shows the formation and maturation of new adhesions occurring under tension-release conditions in cells expressing active vinculin. Video 2 shows that Mn^2+^-activated integrins in talinKO cells support talin-independent adhesion formation.
